# Contrast-induced nephropathy and oxidative stress: mechanistic insights for better interventional approaches

**DOI:** 10.1186/s12967-020-02574-8

**Published:** 2020-10-20

**Authors:** Prit Kusirisin, Siriporn C. Chattipakorn, Nipon Chattipakorn

**Affiliations:** 1 Division of Nephrology, Department of Internal Medicine, Faculty of Medicine, Chiang Mai University, Chiang Mai, Thailand; 2 Cardiac Electrophysiology Research and Training Center, Faculty of Medicine, Chiang Mai University, Chiang Mai, Thailand; 3 Center of Excellence in Cardiac Electrophysiology Research, Chiang Mai University, Chiang Mai, Thailand; 4 Cardiac Electrophysiology Unit, Department of Physiology, Faculty of Medicine, Chiang Mai University, Chiang Mai, Thailand

**Keywords:** Contrast-induced nephropathy, Oxidative stress, Mitochondria, Prevention, Statin

## Abstract

Contrast-induced nephropathy (CIN) or contrast-induced acute kidney injury (CI-AKI) is an iatrogenic acute kidney injury observed after intravascular administration of contrast media for intravascular diagnostic procedures or therapeutic angiographic intervention. High risk patients including those with chronic kidney disease (CKD), diabetes mellitus with impaired renal function, congestive heart failure, intraarterial intervention, higher volume of contrast, volume depletion, old age, multiple myeloma, hypertension, and hyperuricemia had increased prevalence of CIN. Although CIN is reversible by itself, some patients suffer this condition without renal recovery leading to CKD or even end-stage renal disease which required long term renal replacement therapy. In addition, both CIN and CKD have been associated with increasing of mortality. Three pathophysiological mechanisms have been proposed including direct tubular toxicity, intrarenal vasoconstriction, and excessive production of reactive oxygen species (ROS), all of which lead to impaired renal function. Reports from basic and clinical studies showing potential preventive strategies for CIN pathophysiology including low- or iso-osmolar contrast media are summarized and discussed. In addition, reports on pharmacological interventions to reduce ROS and attenuate CIN are summarized, highlighting potential for use in clinical practice. Understanding this contributory mechanism could pave ways to improve therapeutic strategies in combating CIN.

## Introduction

Contrast-induced nephropathy (CIN) or contrast-induced acute kidney injury (CI-AKI) is an iatrogenic acute kidney injury (AKI) observed after intravascular administration of contrast media (CM) for diagnostic procedures or therapeutic angiographic interventions [1–4]. Chemical hypersensitivity has also been reported as another side effect of CM [5]. According to the Kidney Disease Improving Global Outcomes (KDIGO) clinical practice guidelines for AKI, a serum creatinine (Cr) increase of at least 0.3 mg/dL (or 26.5 µmol/L) over the baseline value within 48 h after exposure to CM, or an increase greater than 1.5 times over the baseline value within 7 days after exposure to CM, or a urinary volume of less than 0.5 mL/kg/h for at least 6 h after exposure, are the definition of this condition [6]. Incidence of CIN has been reported in 1–25% of cases of hospital-acquired AKI, and is the third common cause of acute tubular necrosis in hospitalized patients leading to prolonged hospitalization [1]. In the general population, the CIN incidence is 1–2% [7]. Although CIN can be reversible, up to 15% of the patients may need temporary dialysis [8]. In patients without renal recovery, CKD can develop 4% progressing to end-stage renal disease (ESRD) [9]. The mortality rate of CIN varies from 3.8 to 64% [10, 11]. Patients with high risk of developing CIN include chronic kidney disease (CKD) and diabetes mellitus (DM) with impaired renal function. Other associated risks include congestive heart failure, volume depletion, old age, hypertension, and hyperuricemia increasing CIN prevalence by up to 25% [7].

**Fig. 1:**
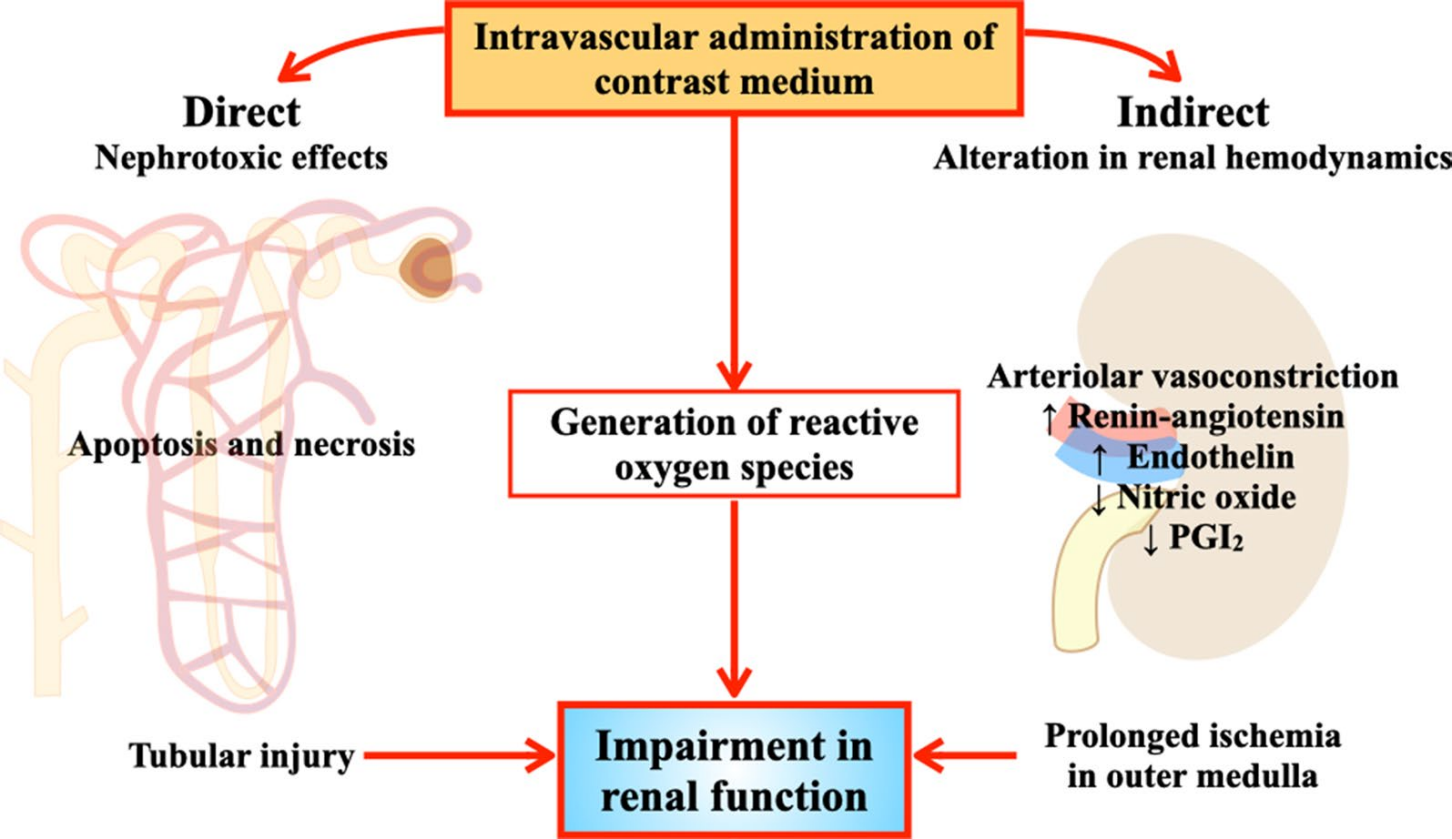
Pathophysiology of CIN. Pathogenesis of CIN consists of 3 mechanisms; direct effect, indirect effect, and generation of ROS. Direct effects include, direct cytotoxicity of CM to nephron leading to cellular apoptosis or necrosis and tubular injury. Indirect effects are that CM could alter renal hemodynamics, leading to intrarenal vasoconstriction, contributing to medullary hypoxia. This mechanism is mediated by the increase in vasoconstrictive mediators including renin, angiotensin II, and endothelin along with the decreasing of vasodilatory mediators including nitric oxide and PGI_2_. Lastly, CM can generate ROS and also reduce antioxidant enzyme activity as a result of various complex mechanisms which result in oxidative stress, leading to progression of impaired renal function. CIN, contrast-induced nephropathy; CM, contrast media; PGI_2_, prostaglandin I_2_; ROS, reactive oxygen species

The pathophysiological mechanisms of CIN have not been completely elucidated. Currently, several mechanisms including direct effect, indirect effect, and generation of reactive oxygen species (ROS) have been proposed (Fig. 1). In direct effects, CM with high osmolality can directly cause cytotoxicity in nephrons including renal tubular epithelial cells and endothelial cells, leading to mitochondrial dysfunction, cellular apoptosis or necrosis and interstitial inflammation [12]. In indirect effects, CM can alter renal hemodynamics, leading to intrarenal vasoconstriction contributing to medullary hypoxia [12]. Regarding ROS generation, CM can either cause excessive ROS production or reduce antioxidant enzyme activity, resulting in increased oxidative stress and leading to impaired renal function [13]. In addition, medullary hypoxia also leads to enhanced ROS formation, resulting in mitochondrial oxidative stress and mitochondrial dysfunction [13]. Overall, it can be seen that mitochondrial function and oxidative stress play important roles in the pathophysiology of CIN [13]. Therefore, strategies that reduce oxidative stress as well as protecting mitochondrial dysfunction are potential targets for CIN prevention.

KDIGO clinical practice guidelines for AKI state there is no definitive treatment available for established CIN [6]. Thus, the prevention of CIN is the best option. This review aims to comprehensively summarize the available in vitro, in vivo, and clinical reports regarding the pathophysiologic roles of mitochondria and ROS in CIN. Reports on pharmacological interventions to prevent CIN by targeting ROS and mitochondria are also presented and discussed with their potential for clinical use in in the future.

## Searching methodology and selection criteria

A comprehensive search of the literature was performed using PubMed covering the period from database inception to September 2019. The search for literature included only articles written in English. An article was rejected if it was clearly a letter or case report. The search used the following keywords: contrast-induced nephropathy; oxidative stress; mitochondria; prevention; and statin either in the title, abstracts, or in the text. The relevance of the subject and eligibility of all publications detected was further evaluated, and data were then extracted from relevant papers to be included in this comprehensive review.

### Pathogenesis of CIN via ROS generation: reports from in vitro studies

In vitro studies offer the unique opportunity to evaluate the activation of intracellular signaling pathways involved in cellular apoptosis or necrosis, which could pave ways for developing specific therapies to be used in in vivo and clinical studies. However, a major shortcoming of preclinical models of CIN relates to the fact that contrast administration alone does not cause AKI in animals. Multiple stressors are required to be utilized concomitantly to inflict CIN; such as inhibition of nitric oxide (NO), dehydration, and use of prostaglandin inhibitor. A summary of findings from in vitro reports is shown in Table 1.

**Table 1:** Roles of oxidative stress in the pathogenesis of contrast-induced nephropathy: reports from in vitro studies

Models	Methods (drug/dose/route/duration)	Major findings			Interpretations	References
		Oxidative stress	Apoptosis	Histopathology		
HK-2 cells	Iohexol/50, 100, 200 mg I/mL/6 h	↑ LDH cell injury in dose-dependent manner	↓ MTT cell viability (100 and 200 mg I/mL) ↑ annexin V-positive cells ↑ mRNA expression of intracellular Nox4 and p22phox	Nuclear fragmentation Organelle reduction Mitochondrial vacuolar degeneration	Iohexol upregulated expression of Nox4 and p22phox, induced cell injury, leading to CIN	[16]
Renal cortical slices isolated from male Fischer 344 (F344) rats	Diatrizoic acid/0, 9.25, 18.5, 37, 74, 111 mg I/mL/60–120 min Iothalamic acid/0, 9.25, 18.5, 37, 74, 111 mg I/mL/60–120 min	↑ LDH leakage in a dose-dependent manner ↔ cellular total GSH ↔ %GSSG	–	–	Diatrizoic acid and iothalamic acid at clinically relevant concentrations caused damage to renal cortical slices	[18]
Human embryonic kidney 293 T cells	Diatriazoate meglumine/11.1 mg I/mL/1, 2, 4, 6 h Iothalamate meglumine/11.1 mg I/mL/1, 2, 4, 6 h Iohexol/11.1 mg I/mL/1, 2, 4, 6 h Iodixanol/11.1 mg I/mL/1, 2, 4, 6 h	–	↑ ATF2 mRNA expression in a time-dependent manner (diatrizoate, iodixanol and iothalamate) ↑ phosphorylation of Thr69/71 of ATF2 in a time-dependent manner (diatrizoate and iothalamate) ↑ phosphorylation of JNK1 and JNK2 in a time-dependent manner (iodixanol, diatrizoate and iothalamate) ↑ cleaved caspase-3 (diatrizoate) ↓ cell viability (diatrizoate and siRNA transfection for ATFz2)	–	Iodinated CM, except iohexol, activated JNK/ATF2 signaling pathways, and diatrizoate caused apoptosis in kidney cells	[19]
HK-2 cells	Iohexol + Nox4 siRNA	↑ Nox2 and Nox4 mRNA expression ↑ ROS production ↓ ROS production (Nox4 siRNA) ↓ GPx and SOD (Nox4 siRNA)	↑ caspase 3/7 activity ↓ caspase 3/7 activity (Nox4 siRNA) ↓ MTT and ATP cell viability ↑ MTT and ATP cell viability (Nox4 siRNA) ↑ MAPK pathways (phospho-p38, JNK and ERK pathways) ↓ phospho-p38, JNK and ERK (Nox4 siRNA) ↑ Bax ↓ Bax (Nox4 siRNA)	–	CM increased ROS production by triggering induction of MAPKs, especially p38 via upregulation of Nox4	[17]
NRK-52E rodent tubular cells	Iohexol/100 mg/mL/3 h + SIRT1 siRNA	↓ SIRT1 ↓↓ SIRT1 in siRNA	↓ MTS cell viability ↓↓ MTS cell viabilitzy in SIRT1 siRNA	–	Iohexol decreased cell viability by downregulation of SIRT1	[21]

ATF2, activating transcriptional factor 2; Bax, Bcl2-associated X protein; Bcl-2, B-cell lymphoma 2; CIN, contrast-induced nephropathy; CM, contrast media; ERK, extracellular signal-regulated kinase; GPx, glutathione peroxidase; GSH, glutathione; GSSG, glutathione disulfide; HK-2 cells, human proximal tubular epithelial cells; HO-1, heme oxygenase 1; HSA-Trx, recombinant human serum albumin-Thioredoxin-1 fusion protein; IV, intravenously; JNK, c-Jun N-terminal kinase; LDH, lactate dehydrogenase; MAPKs, mitogen-activated protein kinases; MESNA, sodium-2-mercaptoethane sulphonate; MTS, 5-(3-carboxymethoxyphenyl)-2H-tetrazolium inner salt; MTT, 3-(4,5-dimethylthiazole-2-yl)-2,5-diphenyltetrazolium bromide; NADPH, nicotinamide adenine dinucleotide phosphate; Nox4, NADPH oxidases; NQO-1, NAD(P)H: quinone oxidoreductase 1; Nrf-2, nuclear factor erythroid 2-related factor 2; p22phox, p22 phagocyte B-cytochrome; PGC-1α, peroxisome proliferator-activated receptor-γ co-activator 1α; ROS, reactive oxygen species; siRNA, short interfering ribonucleic acid; SIRT1, sirtuin 1; SOD, superoxide dismutase

### Roles of mitogen-activated protein kinase (MAPK) pathways in CIN

ROS induced by CM could activate the MAPK signaling pathway through 4 cascades, including extracellular signal-related kinases (ERK) 1 and 2, c-JUN N-terminal kinase (JNK) 1, 2, and 3, p38-MAPK, and ERK5 [14]. These pathways contribute to the activation of caspase-9 and caspase-3, thus inducing apoptosis [15]. In HK-2 cells, CM increased cell injury and decreased cell viability, leading to severe mitochondrial vacuolar degeneration and nucleus fragmentation [16]. CM also increased ROS production via upregulation of nicotinamide adenine dinucleotide phosphate oxidase 2 (Nox2), Nox4 and p22phox, [16] and triggered apoptosis via induction of caspase 3/7 activity, MAPK pathways (including p38, JNK and ERK pathways), and B-cell lymphoma 2-associated X protein (Bax) expression [17]. Transfection of Nox4 short interfering ribonucleic acid (siRNA) caused a reduction in ROS production and apoptosis [17]. These findings indicated that both Nox and MAPK pathways are involved in the CM-induced ROS production.

Different types of high-osmolar CM were studied in renal cortical cells isolated from male Fischer 344 rats (Table 1). CM was shown to induce renal cell injury in a dose-dependent manner regardless of type of high-osmolar CM [18]. In human embryonic kidney 293 T cells, CM activated JNK/activating transcriptional factor 2 (ATF2) signaling pathways and decreased cell viability. Transfection with ATF2 siRNA caused reduced apoptosis in those CM-treated cells [19]. These findings indicated that JNK/ATF2 pathways are involved in CM-induced ROS production (Table 1).

### Roles of silent information regulator 1 (SIRT1) in CIN

SIRT1 is a histone deacetylase of nicotinamide adenine dinucleotide (NAD^+^), which mainly exists in the nucleus [20]. In NRK-52E rodent tubular cells, CM caused oxidative stress and decreased cell viability by downregulation of SIRT1 [21]. Transfection with SIRT1 siRNA resulted in increased apoptosis in these cells treated with CM [21]. These findings indicated that CM downregulated SIRT1, leading to increased cell apoptosis (Table 1).

## Pathogenesis of CIN via ROS generation: reports from in vivo studies

Consistent with in vitro reports, data from in vivo studies demonstrated that CM increased ROS levels and apoptosis, leading to impaired renal function [19]. A summary of these in vivo reports is shown in Table 2.

**Table 2:** Roles of oxidative stress in pathogenesis of contrast-induced nephropathy: reports from in vivo studies

Animals	Models	Major findings					Interpretations	References
		Renal function	Oxidative stress	Inflammatory markers	Apoptosis	Histopathology		
Male BALB/c mice	Restricted water 24 h treated with iodixanol/IV/24 h	↑ Cr ↑ BUN ↑ urinary NAG ↓ RBF	↑ ROS ↑ 8-OHdG-positive cells ↓ SOD-1 ↔ SOD-2	↑ phospho- NF-*k*B p65 ↑ TNF-α ↑ IL-6 ↑ iNOS-positive cells	↑ ROCK-2 protein ↑ p-MYPT1 and p-MYPT1/MYPT ratio ↑ TUNEL-positive cells ↑ cleaved caspase-3 ↑ Bax ↓ p-Akt/total Akt ratio	Moderate tubular injury with tubular degeneration Loss of brush border membranes Formation of cast Vacuolization of tubular epithelial cells Dilation of tubules	Iodixanol increased ROCK-2 activity, contributing to increased NF-*k*B transcriptional activity, oxidative stress, inflammation and apoptosis, leading to impaired renal function	[23]
Male C57BL/6 J mice	L-NAME/IP + indomethacin/IP treated with iohexol/IP/1 h	↑ Cr	↓ SIRT1 ↑↑ PGC-1α expression ↑ phosphor-Ser^256^ FoxO1 expression ↓ SOD2 ↑ MDA	–	↑ TUNEL-positive cells ↑ cleaved caspase-3	Tubular vacuolization Disruption of tubular structures in outer medulla ↑ macrophage infiltration	Iohexol upregulated SIRT1-PGC-1α-FoxO1 signaling mediated oxidative stress, apoptosis, leading to impaired renal function	[21]
Male Sprague–Dawley rats	Dehydration 48 h treated with iohexol/IV/24 h	↑ Cr ↑ BUN	↑ 8-OHdG-positive cells ↑ MDA	–	↑ TUNEL-positive cells ↑ Nrf-2-positive cells ↑ p-Akt/Akt ↑ nuclear-Nrf-2 ↑ HO-1/Actin	Severe tubular detachment Foamy degeneration of tubular cells	CM upregulated PI3K/Akt/Nrf-2 pathway, leading to increased oxidative stress and apoptosis, leading to impaired renal function	[26]
Adult Sprague Dawley rats	Indomethacin/IV + L-NAME/IV treated with ioversol/IV/72 h	↑ Cr ↑ BUN	↑ MDA ↓ SOD	–	↑ Nrf-2/HO-1-positive cells ↑ HO-1-positive cells ↑ Nrf-2, NQO-1 and HO-1 gene expression ↑ Nrf-2 nuclear translocation ↑ HO-1 and NQO-1 protein levels	Tubular necrosis Hemorrhagic casts	Nrf-2/HO-1 pathway regulated adaptive cytoprotective responses to counteract tissue damage, oxidative stress and apoptosis caused by CM	[27]
Male Sabra rats (Wistar-derived colony)	Low sodium diet 7 d + indomethacin/IV treated with iothalamate/IV	↑ Cr ↓ CrCl	↑ O_2_^−^ production	↓ eNOS ↑ iNOS	↑ HO-1 protein ↑ renal heme ↑ caspase-3 ↑ caspase-9 ↑ Bax ↓ Bcl-2	–	Increased level of HO-1 are protective against AKI due to CM exposure	[28]
Male C57BL/6 mice	Water deprivation 16 h + indomethacin/IP + L-NAME/IP treated with iohexol/24 h	↑ BUN ↔ Cr ↑ KIM-1-positive cells	↔ SOD ↔ Nox4 ↔ Nox1 ↑ Nox2 ↑ 8-OHdG-positive cells	–	↑ phospho-p38/p38 ↑ phospho-pJNK/pJNK ↑ phospho-ERK/ERK ↑ Bax ↓ Bcl-2 ↑ TUNEL-positive cells	Tubular epithelial cell shedding Basement membrane nudity Vacuolar degeneration of tubular epithelial cells Protein casts Tubular dilation Loss of tubular brush borders Necrosis of partial tubular epithelial cells ↑ tubular pathological scores	The Nox4/Nox2 axis was involved in the amplification of ROS production, apoptosis and CIN progression, leading to impaired renal function	[17]
Male Wistar rats	Diatrizoate/no dose provided/IV/1, 24 h	–	–	–	↑ TUNEL-positive cells	–	Diatrizoate caused apoptosis, leading to impaired renal function	[19]

AKI, acute kidney injury; Bax, Bcl2-associated X protein; Bcl-2, B-cell lymphoma 2; CAG, coronary angiography; CIN, contrast-induced nephropathy; CM, contrast media; Cr, creatinine; CrCl, creatinine clearance; DNA, deoxyribonucleic acid; eNOS, endothelial nitric oxide; HO-1, heme oxygenase 1; iNOS, inducible nitric oxide synthase; KIM-1, kidney injury molecule-1; L-NAME, N^ω^-nitro-L-arginine methyl ester; MDA, malondialdehyde; MESNA, sodium-2-mercaptoethane sulphonate; NADPH, nicotinamide adenine dinucleotide phosphate; NF-*k*B, nuclear factor-*k*B; NO, nitric oxide; Nox4, NADPH oxidases; NQO-1, NAD(P)H: quinone oxidoreductase 1; Nrf-2, nuclear factor erythroid 2-related factor 2; ROS, reactive oxygen species; TUNEL, terminal deoxynucleotide transferase dUTP nick end labeling; 8-OHdG, 8-hydroxy-2′-deoxyguanosine

### Roles of MAPK and SIRT1 in CIN

In mice, CM administration activated the Nox4/Nox2 axis, resulting in increased ROS production, and involving the MAPK pathway (including p38, JNK and ERK pathways) resulting in apoptosis, leading to impaired renal function [17]. CM administration also downregulated SIRT1 and upregulated peroxisome proliferator-activated receptor gamma-assisted activating factor-1α-Forkhead-box transcription factor 1 (PGC-1α-FoxO1) signaling mediated oxidative stress and apoptosis, leading to impaired renal function (Table 2) [21].

### Roles of Rho/Rho-kinase (Rho/ROCK) pathway in CIN

The Rho/ROCK pathway is an important regulator in vascular smooth muscle cell contraction, cell migration, proliferation and differentiation [22]. Administration of CM in mice increased Rho/ROCK pathway activity, contributing to increased nuclear factor-*k*B (NF-*k*B) transcriptional activity, oxidative stress, inflammation and apoptosis, finally resulting in impaired renal function (Table 2) [23].

### Roles of nuclear factor erythroid 2-related factor 2/heme oxygenase 1 (Nrf-2/HO-1) pathway in CIN

**Fig. 2:**
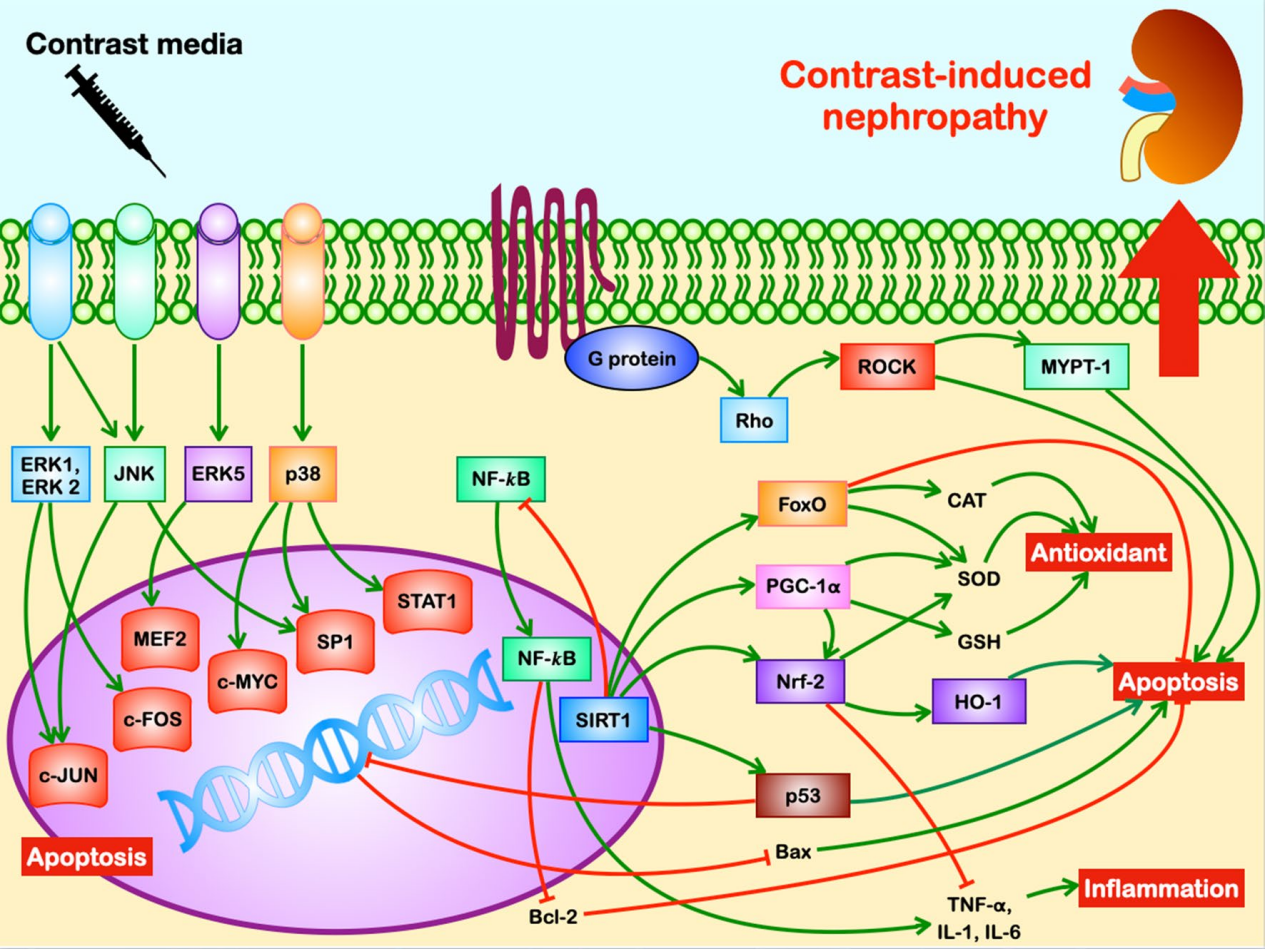
Mechanism of CIN via complex pathways of ROS from in vitro and in vivo studies. Contrast media can generate ROS especially in high risk patients such as DM and CKD through 4 major pathways: (1) MAPK pathway including ERK, JNK and p38; (2) SIRT1 pathway including SIRT1, FoxO, NF-*k*B, PGC-1 and p53; (3) Rho/ROCK pathway including MYPT-1 and NF-*k*B; (4) Nrf-2/HO-1 pathway including Nrf-2, NQO1, GSH and HO-1. CIN, contrast-induced nephropathy; CKD, chronic kidney disease; DM, diabetes mellitus; ERK, extracellular signal-related kinases; FoxO, Forkhead-box transcription factor; GSH, glutathione; JNK, c-JUN N-terminal kinase; MAPK, mitogen-activated protein kinase; MYPT-1, myosin-phosphatase target unit; NF-*k*B, nuclear factor-*k*B; NQO1, nicotinamide adenine dinucleotide phosphate quinone oxidoreductase 1; Nrf-2/HO-1, nuclear factor erythroid 2-related factor 2/heme oxygenase 1; PGC-1, peroxisome proliferator-activated receptor gamma-assisted activating factor-1; ROCK, rho-kinase; ROS, reactive oxygen species; SIRT1, silent information regulator 1

The Nrf-2/HO-1 pathway is involved in many functions including mitochondrial oxidative stress, autophagy, and programmed cell death [24]. Nrf-2, when translocated into the nucleus, stimulates transcription of genes that encode detoxifying and antioxidant enzymes, contributing to cellular protection by reducing oxidative stress [25]. In CM-treated rats, the Nrf-2/HO-1 pathway was upregulated to develop adaptive cytoprotective responses to counteract tissue damage, increased oxidative stress and apoptosis caused by CM (Table 2) [26–28]. Fig. 2 illustrates the mechanisms involved in the pathogenesis of CIN from in vitro and in vivo reports.

## Interventions targeting ROS for CIN prevention: evidence from in vitro reports

In HK-2 cells [16] and MDCK cells, [29] atorvastatin attenuated CM-induced cytotoxicity through the downregulation of Nox4 and p22phox, and activation of MAPK pathways via JNK and tumor suppressor p53 activation [16, 29]. GKT137831, a specific Nox1/4 inhibitor, also decreased Nox2 expression, leading to decreased ROS production, and reduced apoptosis via decreasing caspase 3/7 activity and Bax along with activating the phosphorylation of p38, JNK and ERK [17].

**Fig. 3:**
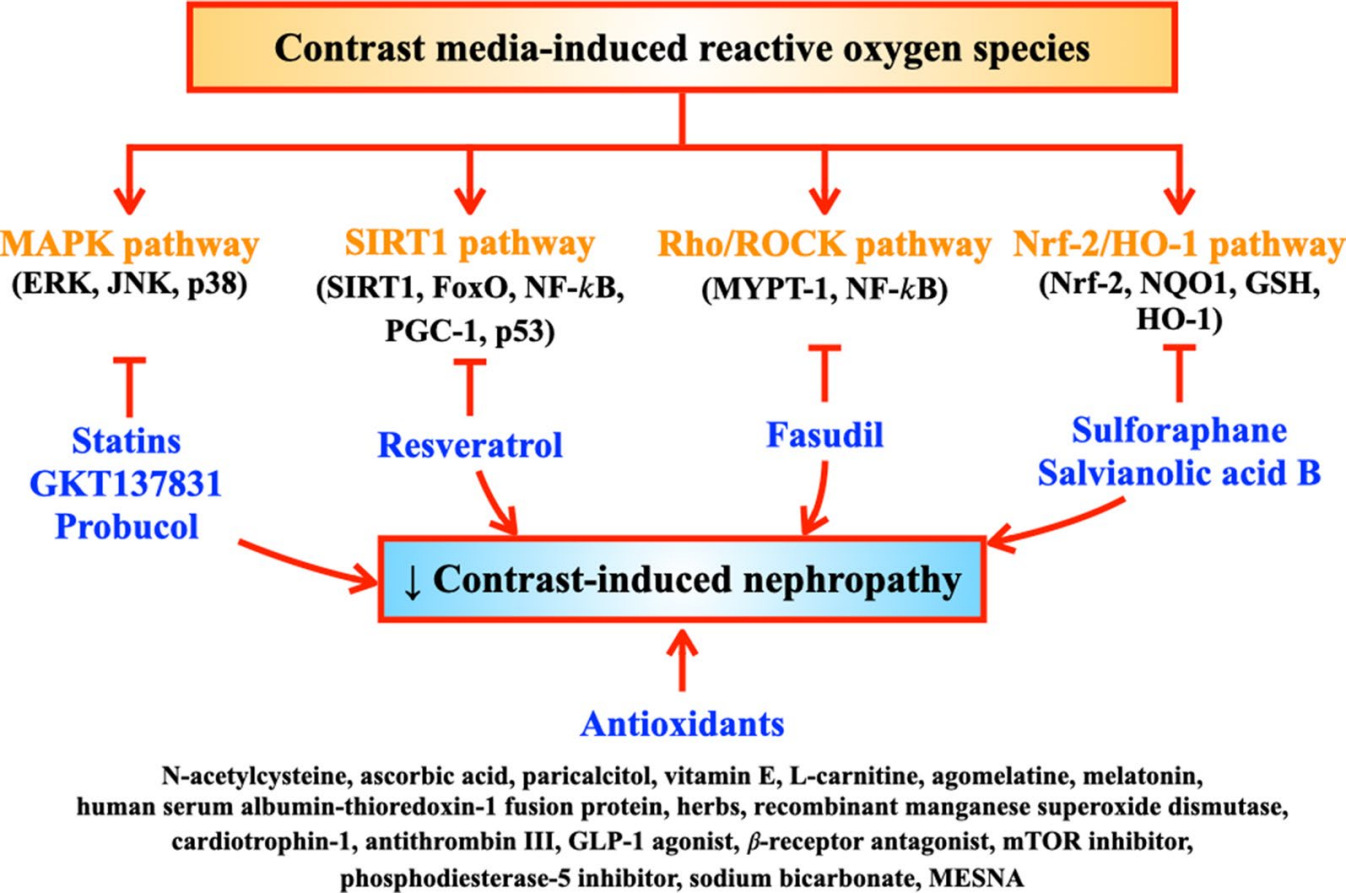
Intervention to reduce ROS for the prevention of CIN: evidence from in vitro, in vivo and clinical studies. In response to the mechanisms involved in ROS production in CIN, interventions to reduce ROS via complex pathways are illustrated. The MAPK pathway was inhibited by statins, GKT137831 and probucol. The SIRT1 pathway was inhibited by resveratrol. Rho/ROCK pathway was inhibited by fasudil. The Nrf-2/HO-1 pathway was inhibited by sulforaphane and salvianolic acid B. Antioxidant agents reported to exert benefits in CIN prevention have also been shown in this figure. CIN, contrast-induced nephropathy; GLP-1, glucagon-like peptide-1; MAPK, mitogen-activated protein kinase; MESNA, sodium-2-mercaptoethane sulphonate; mTOR, mammalian target of rapamycin; Nrf-2/HO-1, nuclear factor erythroid 2-related factor 2/heme oxygenase 1; ROCK, rho-kinase; ROS, reactive oxygen species; SIRT1, silent information regulator

Resveratrol, a known SIRT1 activator, was shown to increase SIRT1, PGC-1α expression, and superoxide dismutase 2 (SOD2), and increased cell viability in NRK-52E rodent tubular cells [21]. These findings indicated that resveratrol attenuated CM-induced nephrotoxicity via activating SIRT1-PGC-1α-FoxO1 signaling, leading to reduced oxidative stress and apoptosis [21]. In addition, Sulforaphane, an Nrf-2 activator, decreased ROS production and increased cell viability in HK-2 cells [27]. These reports are summarized in Table 3 and Fig. 3.

**Table 3:** Interventions to attenuate oxidative stress in contrast-induced nephropathy: reports from in vitro studies

Models	Methods (drug/dose/route/duration)	Major findings			Interpretations	References
		Oxidative stress	Apoptosis	Histopathology		
HK-2 cells treated with iohexol/200 mg I/mL/6 h	Atorvastatin/1, 20, 40 µM/2 h prior to iohexol	–	↑ MTT cell viability ↓ annexin V-positive cells ↓ mRNA expression of intracellular NOX4 and p22phox All effects by atorvastatin 40 µM	↓ nuclear fragmentation ↓ organelle reduction ↓ mitochondrial vacuolar degeneration	Atorvastatin attenuated iohexol-induced cytotoxicity through downregulation of NOX4 and p22phox	[16]
MDCK cells & HK-2 cells treated with iodixanol/200 mg/mL/3 h	Atorvastatin/0.2 µmol/L/12 h prior to iodixanol	–	↑ MTS cell viability ↓ caspase-3 ↓ JNK ↓ p53 phosphorylation	–	Pretreatment with atorvastatin reduced contrast-induced JNK activation, leading to apoptosis	[29]
HK-2 cells treated with iohexol 150 mg I/mL/12 h	Specific Nox1/4 inhibitor (GKT137831)/20 µg/mL/30 min prior to iohexol	↑ Nox2 and Nox4 mRNA expression ↓ ROS production	↓ caspase 3/7 activity ↑ MTT and ATPlite cell viability ↓ MAPK pathways (phospho-p38, JNK and ERK pathways) ↓ Bax	–	Inhibition of Nox4 activity attenuated CIN	[17]
NRK-52E rodent tubular cells treated with iohexol/100 mg/mL/3 h	Resveratrol/10, 50 µmol/24 h prior to iohexol	↑ SIRT1 ↑ PGC-1α expression ↑ SOD2	↑ MTT cell viability	–	Resveratrol attenuated iohexol-induced nephrotoxicity via activating SIRT1-PGC-1α-FoxO1 signaling, leading to reduced oxidative stress and apoptosis	[21]
HK-2 cells treated with ioversol/50 mg/mL/24 h	Sulforaphane (Nrf-2 activator)/5 µmol/L/30 min prior to ioversol	↓ ROS production	↑ MTT cell viability ↑ Nrf-2, NQO-1 and HO-1 gene expression ↔ MTT cell viability in Nrf-2 siRNA	–	Renoprotective effect of sulforaphane in ioversol-induced nephrotoxicity was associated with Nrf-2/HO-1 pathway	[27]
HK-2 cells treated with H_2_O_2_/250 µM/L/3, 24 h	HSA-Trx/0.1, 0.5, 1, 5, 10 µmol/L/1 h prior to H_2_O_2_	↓ ROS production in a dose-dependent manner	↓ WST-8-positive cells in a dose-dependent manner	–	HSA-Trx attenuated oxidative stress and inflammation in CIN	[37]
HK-2 cells treated with H_2_O_2_/500 µmol/L/24 h	Magnolin/10, 40 µg/mL/prior to H_2_O_2_	↓ ROS	↓ caspase-3 ↑ Bcl-2	–	Magnolin attenuated oxidative stress and apoptosis	[49]
HK-2 cells treated with H_2_O_2_/250 mM/3, 24 h	Salvianolic acid B/50 µM/1 h prior to H_2_O_2_ Wortmannin (PI3K inhibitor)/10 µM/1 h prior to H_2_O_2_	↓ ROS production	↑ MTT cell viability ↑ CCK-8 cell viability ↑ p-Akt and nuclear-Nrf-2 expression (salvianolic acid B) ↓ p-Akt and nuclear-Nrf-2 expression (wortmannin)	–	Salvianolic acid B attenuated oxidative stress and provided cell protection via PI3K/Akt/Nrf-2 pathway	[26]

ATF2, activating transcriptional factor 2; Bax, Bcl2-associated X protein; Bcl-2, B-cell lymphoma 2; CIN, contrast-induced nephropathy; ERK, extracellular signal-regulated kinase; GPx, glutathione peroxidase; GSH, glutathione; GSSG, glutathione disulfide; HK-2 cells, human embryonic proximal tubular epithelial cells; HO-1, heme oxygenase 1; HSA-Trx, recombinant human serum albumin-Thioredoxin-1 fusion protein; IV, intravenously; JNK, c-Jun N-terminal kinase; LDH, lactate dehydrogenase; MAPKs, mitogen-activated protein kinases; MDCK cells, Madin Darby distal nonhuman tubular epithelial cells; MESNA, sodium-2-mercaptoethane sulphonate; MTS, 5-(3-carboxymethoxyphenyl)-2H-tetrazolium inner salt; MTT, 3-(4,5-dimethylthiazole-2-yl)-2,5-diphenyltetrazolium bromide; NADPH, nicotinamide adenine dinucleotide phosphate; Nox4, NADPH oxidases; NQO-1, NAD(P)H: quinone oxidoreductase 1; Nrf-2, nuclear factor erythroid 2-related factor 2; p22phox, p22 phagocyte B-cytochrome; PGC-1α, peroxisome proliferator-activated receptor-γ co-activator 1α; ROS, reactive oxygen species; siRNA, short interfering ribonucleic acid; SIRT1, sirtuin 1; SOD, superoxide dismutase

## Interventions targeting ROS for CIN prevention: evidence from in vivo reports

### Interventions targeting MAPK, SIRT1, Rho/ROCK and Nrf-2/HO-1 pathways

In mice, GKT137831 could ameliorate oxidative stress via increased SOD and decreased Nox2, reducing apoptosis through the phosphorylation of p38, JNK, ERK, thus resulting in improved renal function [17]. Resveratrol was shown to attenuate CIN in rats via increasing SIRT1, PGC-1α, and SOD2, and decreasing phosphorylation of Ser^256^ FoxO1 expression, leading to a reduction in oxidative stress, apoptosis, improving renal function [21].

Fasudil, a Rho kinase inhibitor, was shown to decrease ROS and increase SOD-1, and reduced Inflammation via the reduction of NF-*k*B p65, interleukin-6 (IL-6), and tumor necrosis factor-α (TNF-α) [23]. Moreover, apoptosis was decreased via a reduction in cleaved caspase-3 and Bax, together with increased B-cell lymphoma-2 (Bcl-2) and p-Akt/total Akt ratio. These benefits on ROS and apoptosis attenuation led to improved renal function [23]. Similarly, sulforaphane was shown to exert CIN protection in rats via the Nrf-2/HO-1 pathway, resulting in reduced renal damage and improved Cr [27]. Salvianolic acid B, a component of Danshen (*Salvia miltiorrhiza* root), attenuated CIN in rats via decreasing malondialdehyde (MDA) and increasing Nrf-2-positive cells, p-Akt/Akt, Nrf-2/Histone H3, and HO-1/Actin, with antioxidative effects through PI3K/Akt/Nrf2 pathway, leading to improved renal function [26]. Table 4 and Fig. 3 show a summary of these reports.

**Table 4:** Interventions to attenuate oxidative stress in contrast-induced nephropathy: reports from in vivo studies

Animals	Models	Intervention (drug/dose/route/duration)	Major findings					Inter-pretations	References
			Renal function	Oxidative stress	Inflammatory markers	Apoptosis	Histo-pathology		
Streptozotocin-induced diabetes in male Wistar rats	Indomethacin/IV + L-NAME/IV + amidotriazoate meglumine	Rosuvastatin/10 mg/kg/day/po/OD/5 day prior to amidotriazoate meglumine	↓ Cr ↑ CrCl ↓ urine microprotein	↓ kidney TBARS ↓ serum MDA ↓ serum PCC ↑ serum thiol	↑ kidney nitrite ↓ IL-6 ↓ TNF-α	↓ TUNEL-positive cells ↓ expression of phospho-p38 ↓ cleaved caspase-3 ↓ Bax/Bcl-2 ratio	↓ histological scores	Rosuvastatin attenuated CIN by modulation of NO, inflammatory responses, oxidative stress and apoptotic processes, leading to improved renal function	[30]
Adult Sprague Dawley rats	Water deprivation 24 h treated with 25% glycerol/IM + iohexol	Simvastatin/15, 30, 60 mg/kg/po/24 h prior to iohexol/4 d	↓ Cr in a dose-dependent manner ↓ BUN in a dose-dependent manner	↓ kidney TBARS ↑ GSH	↓ MPO ↑ NO	–	↓ tubular dilatation, tubular vacuolation, and tubular necrosis in a dose dependent manner	Simvastatin prevented CIN and structural changes in kidney via a reduction of oxidative stress and inflammation, leading to improved renal function	[32]
Male Sprague–Dawley rats	Water deprivation 72 h + furosemide 10 mg/kg/IM treated with iohexol	Rosuvastatin/10 mg/kg/day/po/OD/3 day before and 4 h after iohexol Simvastatin/80 mg/kg/day/po/OD/3 day before and 4 h after iohexol Atorvastatin/20 mg/kg/day/po/OD/3 day before and 4 h after iohexol	↓ Cr by atorvastatin and rosuvastatin	↓ kidney TBARS ↓ serum MDA ↑ serum thiol	↓ IL-6 ↓ MCP-1 ↓ TNF-α Most effective in rosuvastatin > atorvastatin ↑ NO by atorvastatin	↓ TUNEL-positive cells ↓ Bax/Bcl-2 ratio Most effective in atorvastatin > rosuvastatin	↓ tubular necrosis and medullary congestion by atorvastatin and rosuvastatin	Atorvastatin and rosuvastatin prevented CIN and reduced oxidative stress In addition, atorvastatin was most effective in attenuating NO system dysfunction and cell apoptosis, whereas rosuvastatin was most effective in reduction of inflammation, leading to improved renal function	[31]
Male Sprague–Dawley rats	Dehydration 3 day treated with furosemide/IM + iohexol/IV	Xuezhikang/2,400 mg/kg/day/po/3 day prior to iohexol Atorvastatin/20 mg/kg/day/po/3 day prior to iohexol	↓ Cr ↓ BUN	↓ renal MDA ↑ GSH	↓ TNF-α ↓ IL-6 ↑ kidney total NO (nitrite/nitrate)	↓ TUNEL-positive cells ↑ Bcl-2/Bax ratio by xuezhikang	↓ tubular necrosis and medullary congestion ↓ medullary damage scores	Xuezhikang and atorvastatin shared similar effect on iohexol-induced CIN, leading to improved renal function	[33]
Female albino Wistar rats	Water deprivation 24 h + 25% glycerol/IM treated with iohexol/IV	Agomelatine/20, 40 mg/kg/po/OD/24 h before and 4 day after iohexol	↓ Cr ↓ BUN	↑ SOD ↑ GSH ↓ MDA	↓ TNF-α ↓ NF-*k*B ↓ IL-6 mRNA expression	–	↓ hyaline and hemorrhagic casts & tubular necrosis	Agomelatine provided nephroprotective, antioxidant and anti-inflammatory effects against CIN in rats, leading to improved renal function	[42]
Adult male Sprague–Dawley rats	Dehydration 24 h + furosemide/IM + indomethacin/IP treated with iomeprol	Melatonin/10 mg/kg/IP/15 min prior to ± 24 h after iomeprol	↓ Cr ↑ CrCl ↓ FENa All effects by pre- and post-treatment	–	–	–	–	Melatonin prevented and attenuated CIN in rats with pre- & post-treatment, leading to improved renal function	[43]
Male Sprague–Dawley rats	Streptozotocin-induced diabetes treated with iohexol/IV	Melatonin/20 mg/kg/day/IP/OD/7 day prior to iohexol	↓ Cr	↓ MDA ↑ SOD ↑ GSH ↓ CAT	↓ MPO ↓ IL-6 ↓ IL-33	–	↓ apoptosis ↓ necrotic changes ↓ glucogenic vacuolization ↓ inflammatory cell infiltration	Melatonin provides functional and histologic protection against CIN via inhibiting of IL-33, leading to improved renal function	[44]
Male Sprague–Dawley rats	Indomethacin/IV + L-NAME/IV treated with ioversol/IV	HSA-Trx/30 mg/kg/IV/1 h prior to ioversol	↓ Cr ↓ BUN ↓ urinary NAG ↑ CrCl	↓ 8-OHdG-positive cells ↓ MDA	–	↓ TUNEL-positive cells	↓ renal tubular injuries	Administration of single dose of HSA-Trx before induction of CIN exerted renoprotective effects in CIN rat model, leading to improved renal function	[37]
Adult male Sprague Dawley rats	Indomethacin/IV + L-NAME/IV treated with iopromide/IV	Vitamin E/250, 500 mg/kg/day/po/5 day prior to iopromide	↓ Cr	↓ MDA ↑ TAC ↑ SOD in a dose-dependent manner	–	–	↓ severity of proximal tubular epithelial cells necrosis and proteinaceous cast ↓ peritubular capillary congestion ↓ interstitial edema	Vitamin E prevented CIN through its antioxidant activity, leading to improved renal function	[35]
Male Sprague–Dawley rats	Indomethacin 10 mg/kg + L-NAME + ioversol/IV	Antithrombin III/500 µg/kg/IV/30 min before or after ioversol	↓ Cr ↓ BUN ↑ renal cortical blood supply ↓ intrarenal resistance index	↓ MDA ↑ SOD	↓ TNF-α ↓ MCP-1 ↓ ICAM-1 expression ↓ F4/80-positive cells infiltration	↓ cleaved caspase-3 expression ↑ Bcl-2	↓ renal tubular detachment ↓ brush border loss ↓ necrosis of tubular cells	Antithrombin III prevented and attenuated CIN through inhibiting inflammation, oxidative stress, apoptosis and improving RBF, leading to improved renal function	[53]
Male Sprague–Dawley rats	Dehydration 72 h treated with iopamidol/IV	Astragaloside IV/20 mg/kg/po/OD/7 day prior to iopamidol	↓ Cr ↓ BUN ↓ cystatin C ↓ NGAL ↓ uKIM-1	↓ MDA ↑ CAT ↑ SOD ↓ serum, urinary and renal 8-OHdG	–	↓ TUNEL-positive cells ↓ caspase-3 activity ↓ cleaved caspase-3 protein expression ↓ Bax protein and mRNA expressions ↑ Bcl-2 protein and mRNA expressions ↓ p38 MAPK phosphorylation	↓ tubular injuries	Astragaloside IV prevented AKI through inhibition of oxidative stress and apoptosis pathways, leading to improved renal function	[45]
Male Wistar rats	Gentamicin/IP/6 day treated with gastrographin/IV	Cardiotrophin-1/100 µg/kg/day/IV/24 h prior to and 4 day after gastrographin	↓ Cr ↓ BUN ↑ CrCl ↑ inulin clearance ↑ RBF ↓ RVR ↓ proteinuria ↓ albuminuria ↓ NAG ↓ uKIM-1 ↓ PAI-1	↓ MDA	–	↓ cleaved caspase-3-positive cells	↓ tubular necrosis in cortex ↓ tubular obstruction with hyaline material in medulla ↓ Ki-67-positive proliferating cells	Cardiotrophin-1 prevented CIN through a reduction of oxidative stress, leading to improved renal function	[54]
Male albino Wistar rats	Water deprivation 24 h + 25% glycerol/IM treated with iohexol/IV	L-carnitine/200, 400 mg/kg/IP/24 h prior to iohexol	↓ Cr ↓ BUN	↑ SOD ↑ GSH ↓ MDA by L-carnitine 400 mg/kg	↓ TNF-α ↓ TGF-1β expression ↓ IL-1β mRNA expression ↓ TNF-α and NF-*k*B-positive cells	↓ caspase-3 mRNA expression	↓ hyaline and hemorrhagic casts ↓ tubular necrosis in cortical segments of proximal tubules	L-carnitine protected against CIN via a reduction of oxidative stress, inflammation and apoptosis in rats, leading to improved renal function	[36]
Male Wistar-albino rats	Dehydration 24 h + furosemide/IM + indomethacin/IP treated with iomeprol/IV	Curcumin/200 mg/kg/day/po/5 day prior to & 5 day after iomeprol	↓ Cr ↓ BUN	↑ SOD ↑ CAT ↑ GSH ↑ GSH-Px ↓ MDA	↓ iNOS-specific-positive cells	↓ LC3/B-specific-positive cells ↓ cleaved caspase 3-specific-positive cells	↓ necrotic and degenerative changes ↓ intertubular hemorrhage	Curcumin attenuated inflammation and apoptosis in CIN, leading to improved renal function	[46]
Male BALB/c mice	Restrict water 24 h treated with iodixanol/IV	Fasudil/3, 10 mg/kg/IV/12, 2 h prior to and 4 h after iodixanol	↓ Cr ↓ BUN ↓ urinary NAG ↑ RBF ↑ renal vasodilation All effects by 10 mg/kg	↓ ROS in a dose-dependent manner ↓ 8-OHdG-positive cells in a dose-dependent manner ↑ SOD-1 ↔ SOD-2	↓ phospho-NF-*k*B p65 ↓ IL-6 ↓ TNF-α ↓ iNOS-positive cells (10 mg/kg)	↓ ROCK-2 protein ↓ p-MYPT1 and p-MYPT1/MYPT1 ratio ↓ TUNEL-positive cells ↓ cleaved caspase-3 ↓ Bax ↑ Bcl-2 ↑ p-Akt/total Akt ratio All effects by 10 mg/kg	↓ tubular injury ↓ formation of cast All effects by 10 mg/kg	Fasudil exerted renoprotective effects by suppressing inflammation, apoptosis and oxidative stress via inhibiting Rho/ROCK pathway and ameliorating hemodynamic disturbances, leading to improved renal function	[23]
Streptozotocin-induced diabetes in male Sprague–Dawley rats	Treated with diatrizoate meglumine/IV	Exendin-4/25 nmol/kg/SC/10 day prior to diatrizoate/11 d	↓ Cr ↓ BUN ↓ urinary albumin excretion ↑ CrCl	↓ MDA ↓ ET-1 ↑ GSH ↑ SOD	↑ nitrate ↑ eNOS	↓ caspase-3 expression	↓ edema ↓ tubular vacuolization ↓ hemorrhage	Pretreatment with exendin-4 ameliorated CIN effects independent of glycemic state, leading to improved renal function	[55]
Female Sprague–Dawley rats	Water deprivation 24 h + diatrizoate/IV	Grape seed proanthocyanidin/100 mg/kg (1 cm^3^)/po/6 day prior to diatrizoate/5 d	↓ Cr ↓ BUN	↓ MDA ↓ TOS ↓ OSI	–	↓ TUNEL-positive cells	↓ perivascular edema ↓ vascular congestion ↓ tubular vacuoles ↓ renal injury score	Proanthocyanidin attenuated CIN by reducing oxidative damage and apoptosis, leading to improved renal function	[47]
Male Wistar albino rats	24-h dehydration + furosemide/IM + indomethacin/IP treated with iomeprol/IV	Lycopene/4 mg/kg/day/po/5 day prior to and 5 day after iomeprol	↓ Cr ↓ BUN	↑ SOD ↑ CAT ↑ GSH ↑ GSH-Px ↓ MDA	↓ iNOS-specific-positive cells	↓ LC3/B-specific positive cells ↓ cleaved caspase 3-specific positive cells	↓ number of infiltrated inflammatory cells and necrotic degenerative changes	Lycopene prevented and attenuated inflammation, autophagy and apoptosis in CIN rats, leading to improved renal function	[48]
Male Sprague–Dawley rats	Indomethacin/IV + L-NAME treated with ioversol/IV	Magnolin/1 mg/kg/SC/15 min prior to ioversol	↓ Cr ↓ BUN ↓ serum NGAL ↓ uKIM-1	↓ MDA ↑ SOD	–	↓ TUNEL-positive cells ↓ caspase-3 activity ↑ Bcl-2 expression	↓ renal tubular injury scores	Magnolin attenuated CIN in rats through reducing oxidative stress and apoptosis, leading to improved renal function	[49]
Male Sprague–Dawley rats	Deprived of water 3 d + indomethacin/IV treated with diatrizoate	Recombinant manganese SOD/15 µg/kg/IP/4 h prior to diatrizoate	↑ GFR	↑ SOD ↓ intrarenal superoxide anion (O_2_^−^) ↓ ROS production	–	–	↓ tubular necrosis ↓ proteinaceous casts	Recombinant manganese SOD reduced oxidative stress, thus preventing CIN, leading to improved renal function	[41]
Adult male Wistar rats	Meglumine ioxaglate/IV	NAC/150 mg/kg/day/IP/6 h before and 6 h after ioxaglate Ozone (5%O_3_ – 95%O_2_)/1 mg/kg/IP/6 h prior to and 6 h after or 5 day after ioxaglate	↓ Cr (NAC) ↓ NGAL	↑ TAC by ozone ↓ PCC	–	–	↓ renal tubular injury ↓ hemorrhage	NAC and ozone treatment prevented and attenuated CIN via a reduction of oxidative stress, leading to improved renal function	[39]
Wistar albino rats	Water deprivation 72 h treated with diatrizoate meglumine/IV	Nebivolol/2 mg/kg/day/po/3 day prior to and 2 day after diatrizoate	↔ Cr ↔ CrCl ↔ BUN ↓ urine microprotein	↓ serum PCC ↓ kidney TBARS ↓ MDA ↑ serum thiol	↑ kidney nitrite levels	–	↓ tubular necrosis ↓ proteinaceous casts ↔ medullary congestion	Nebivolol attenuated either systemic or renal oxidative stress and increased either nitrite production or restored pathology, leading to improved renal function	[56]
Male Wistar albino rats	Indomethacin/IV + L-NAME/IV treated with amidotrizoate meglumine/IV	Paricalcitol/0.4 µg/kg/day/IP/3 day prior to and 2 day after amidotrizoate	↓ Cr ↑ CrCl ↓ FENa	↓ MDA ↓ kidney TBARSs	↓ VEGF score	–	↓ tubular necrosis ↓ proteinaceous casts ↓ medullary congestion	Paricalcitol reduced unfavourable histopathology of CIN via antioxidant effects, leading to improved renal function	[38]
Male Sprague Dawley rats	Indomethacin/IV + L-NAME/IV treated with iopromide/IV	*Phyllanthus emblica* extract/125, 250, 500 mg/kg/day/po/5 day prior to iopromide	↓ Cr (250, 500 mg/kg/d) ↓ BUN	↓ MDA (250, 500 mg/kg/d) ↑ TAC (250, 500 mg/kg/d) ↑ SOD ↑ CAT	–	–	↓ tubular necrosis ↓ proteinaceous cast formation ↓ peritubular capillary congestion ↓ interstitial edema All changes by 250, 500 mg/kg/d	*Phyllanthus emblica* extract exerted renoprotective effects of CIN in rat, leading to improved renal function	[50]
Streptozotocin-induced diabetes in male Sprague–Dawley rats	Treated with diatrizoate/IV	5% Probucol/500 mg/kg/po/14 day prior to diatrizoate	↓ Cr ↑ CrCl	–	–	↑ p-ERK1/2 ↓ p-JNK ↑ Bcl-2 ↓ Bax ↓ caspase-3	↓ vacuolar degeneration of renal tubular cells ↑ dilatation of lumen ↓ renal tubular injury score	Probucol exerted protective effects on CIN in diabetic rats via inhibition of renal cell apoptosis, leading to improved renal function	[34]
Male Sprague–Dawley rats	Iohexol/IP	Rapamycin/2, 5 mg/kg/IP/7 day prior to iohexol	↓ Cr in a dose-dependent manner	↓ MDA in a dose-dependent manner ↓ CAT in a dose-dependent manner	–	↑ LC3II/I ↑ Beclin-1 ↑ Pink1 ↓ P62 ↑ ∆ψm in a dose-dependent manner ↓ cytosolic/mitochondrial Cyt c in a dose-dependent manner ↑ TOMM20-stained mitochondria in a dose-dependent manner ↑ LC3-stained autophagosomes ↑ LAMP2-stained lysosomes ↓ renal tubular epithelial cell apoptosis in a dose-dependent manner	↓ renal tubular necrosis in a dose-dependent manner	Rapamycin exerted renoprotective effects against CIN via suppressing mitochondrial injury and oxidative stress, mitophagy and apoptosis, leading to improved renal function	[62]
Male C57BL/6 J mice	L-NAME/IP + indomethacin/IP treated with iohexol/IP	Resveratrol/30 mg/kg/IP/simultaneously with iohexol	↓ Cr	↑ SIRT1 ↑ PGC-1α expression ↓ phosphor-Ser^256^ FoxO1 expression ↑ SOD2 ↓ MDA	–	↓ TUNEL-positive cells ↓ cleaved caspase-3	↓ severity score for tubular vacuolization ↓ disruption of tubular structures ↓ macrophage infiltration	Resveratrol attenuated CIN via a reduction of oxidative stress and apoptosis, leading to improved renal function	[21]
Wistar rats	Indomethacin/IV + L-NAME/IV treated with diatrizoate meglumine/IV	NAC/100 mg/kg/po/7 day prior to diatrizoate Salidroside/20 mg/kg/IP/7 day prior to diatrizoate	↓ Cr ↓ BUN ↓ NAG ↓ 24-h urinary protein	↑ SOD ↓ MDA ↓ angiotensin II ↓ 8-OHdG	↑ NO ↑ eNOS mRNA and protein ↑ NOS activity	–	↓ disintegrated and shed brush border of tubular epithelial cells ↓ vacuolar degeneration ↓ cell debris and protein cast in tubular lumen ↓ focal interstitial edema and inflammatory cell infiltration	Salidroside or NAC prevented CIN via a reduction of oxidative stress, leading to improved renal function	[40]
Male Sprague–Dawley rats	Dehydration 48 h treated with iohexol/IV	Salvianolic acid B/50 mg/kg/IV/5 min prior to iohexol Wortmannin (PI3K inhibitor)/15 µg/kg/IV/5 min prior to iohexol Sulforaphane (Nrf-2 activator)/10 mg/kg/IV/5 min prior to iohexol	↓ Cr (salvianolic acid and sulforaphane) ↓ BUN (salvianolic acid) ↑ Cr (wortmannin)	↓ 8-OHdG-positive cells (salvianolic acid and sulforaphane) ↔ 8-OHdG-positive cells (wortmannin) ↓ MDA (salvianolic acid and sulforaphane) ↔ MDA (wortmannin)	–	↓ TUNEL-positive cells (salvianolic acid and sulforaphane) ↑ TUNEL-positive cells (wortmannin) ↑ Nrf-2-positive cells (salvianolic acid and sulforaphane) ↔ Nrf-2-positive cells (wortmannin) ↑ p-Akt/Akt (salvianolic acid) ↔ p-Akt/Akt (sulforaphane) ↓ p-Akt/Akt (wortmannin) ↑ Nrf-2/Histone H3 (salvianolic acid and sulforaphane) ↓ Nrf-2/Histone H3 (wortmannin) ↑ HO-1/Actin (salvianolic acid and sulforaphane) ↓ HO-1/Actin (wortmannin)	↓ histological scores (tubular epithelium degeneration) (salvianolic acid B and sulforaphane) ↑ histological scores (wortmannin)	Salvianolic acid B exerted renoprotection and antioxidative effects through PI3K/Akt/Nrf2 pathway, leading to improved renal function	[26]
Male Sprague–Dawley rats	Gentamicin/SC + iothalamate meglumine/IV	Sesame oil/0.5 ml/kg/po/1 h prior to iothalamate	↓ Cr ↓ BUN	↓ MDA ↓ renal hydroxyl radicals ↓ renal superoxide anion generation	↓ MPO ↓ renal nitrite/nitrate level ↓ iNOS expression	–	↓ inflammatory cell infiltration ↓ tubular dilation ↓ congestion in tubules	Sesame oil prevented CIN via inhibiting oxidative stress in rats, leading to improved renal function	[51]
Male Wistar rats	24-h water deprivation + L-NAME/IP + indomethacin/IP treated with iohexol/IV	Sildenafil citrate/50 mg/kg/day/po/5 day prior to and 2 day after iohexol	↓ Cr ↑ GFR ↑ RPF ↑ RBF ↓ RVR ↓ BUN ↓ proteinuria	↓ intracellular O_2_^−^ ↓ H_2_O_2_	–	–	–	Sildenafil prevented CIN through vasodilator and antioxidant activity, leading to improved renal function	[58]
Male Wistar rats	12-h dehydration + L-NAME/IP + indomethacin/IP treated with iopromide/IV	Sildenafil/10 mg/kg/day/po/7 day prior to iopromide Taladafil/5 mg/kg/day/po/7 day prior to iopromide NAC/100 mg/kg/day/po/7 day prior to iopromide	↓ Cr ↓ BUN	–	–	–	↓ hydropic changes of renal tubules ↓ Bowman space with lobulated glomerulus ↓ alteration of macula densa	Sildenafil and taladafil prevented CIN-related structural kidney damage and superior to NAC	[59]
Male Wistar rats	12-h dehydration + L-NAME/IP + indomethacin/IP treated with iopromide/IV	Sildenafil/10 mg/kg/day/po/7 day prior to iopromide Taladafil/5 mg/kg/day/po/7 day prior to iopromide NAC/100 mg/kg/day/po/7 day prior to iopromide	↓ Cr ↓ BUN	↑ TAC ↑ GSH ↑ CAT ↓ PCC ↓ TBARS	–	–	–	Sildenafil and taladafil prevented CIN through antioxidant activity	[60]
Adult male Swiss mice	Overnight water deprivation + L-NAME/IP + indomethacin/IP treated with ioversol/IP	NAC/200 mg/kg/po/5 day prior to ioversol Silymarin/50, 200, 300 mg/kg/po/5 day prior to ioversol	↓ Cr in a dose-dependent manner (silymarin) ↓ BUN in a dose-dependent manner ( silymarin) ↓ cystatin C in a dose-dependent manner (silymarin)	↓ intracellular superoxide (O_2_^−^) ↓ H_2_O_2_ ↓ OH^−^/ONOO^−^ ↓ advanced oxidation protein products in plasma (silymarin 300 mg)	–	↓ DNA damage (silymarin 300 mg) ↓ annexin V-positive cells	↓ shrunken glomerular tuft ↓ loss of structural cohesion with atypical podocytes ↓ loss of nuclei ↓ tubular dilation with luminal congestion ↓ tubular epithelial cell vacuolization ↓ tubular shedding ↓ tubulo-interstitial lesions	Silymarin decreased systemic and renal oxidative damage, preserving renal function, morphological architectures antigenotoxic and antiapoptotic activities under exposure to radiocontrast agent in mice, leading to improved renal function	[52]
Adult Wistar Albino rats	Iodixanol/IV	Sphingosylphosphorylcholine/2, 10 µM/IP/3 day after iodixanol	↔ Cr ↓ BUN	↑ SOD ↓ MDA	↓ NO ↓ iNOS-positive cells	↓ TUNEL-positive cells	↓ widespread loss of brush border ↓ denudation of tubular cells ↓ tubule dilatation ↓ intratubular obstruction by granular casts	Sphingosyl-phosphoryl-choline reduced CIN via preventing oxidative stress and apoptosis, leading to improved renal function	[63]
Adult Sprague Dawley rats	Indomethacin/IV + L-NAME/IV treated with ioversol/IV	Sulforaphane/5 mg/kg/po/5 day prior to ioversol	↓ Cr ↓ BUN	↓ MDA ↑ SOD	–	↑ Nrf-2, NQO-1 and HO-1 gene expression ↑ Nrf-2 nuclear translocation ↑ HO-1 and NQO-1 protein levels	↓ tubular necrosis ↓ hemorrhagic casts	Sulforaphane ameliorated CIN via Nrf-2/HO-1 pathway, leading to improved renal function	[27]
Male C57BL/6 mice	Water deprivation 16 h + indomethacin/IP + L-NAME/IP treated with iohexol	GKT137831 (Nox1/4 inhibitor)/40 mg/kg/po/5 day prior to iohexol	↔ Cr ↓ BUN ↓ KIM-1-positive cells	↑ SOD ↔ Nox4 ↔ Nox1 ↓ Nox2 ↓ 8-OHdG-positive cells	–	↓ phospho-p38/p38 ↓ phospho-pJNK/pJNK ↓ phospho-ERK/ERK ↓ Bax ↑ Bcl-2 ↓ TUNEL-positive cells	↔ tubular epithelial cell degeneration ↓ basement membrane nudity ↓ vacuolar degeneration of tubular epithelial cells ↓ protein casts ↓ tubular dilation ↓ loss of tubular brush borders ↓ necrosis of partial tubular epithelial cells ↓ tubular pathological scores	Inhibition of Nox1/4 prevented CIN via a reduction of oxidative stress and apoptosis, leading to improved renal function	[17]
Male Wistar albino rats	Dehydration 3 day treated with diatrizoate/IV	Carvedilol/2 mg/kg/po/3 day prior to diatrizoate Nebivolol/2 mg/kg/po/3 day prior to diatrizoate	↔ Cr ↔ BUN	↓ MDA ↑ TAC ↔ SOD	–	–	↓ interstitial inflammation ↓ tubular degeneration ↓ tubular dilatation	Both carvedilol and nebivolol attenuated oxidative stress but did not improve renal function	[57]
Female Wistar albino rats	Furosemide/SC + deprived of water for 24 h treated with iothalamate sodium/IV	8.4% NaHCO3/1 mL/IV/3 h prior to iothalamate	↔ Cr ↔ CrCl	↔ MDA ↓ GSH	↔ MPO ↔ NO	–	↓ % of tubular injury	Urinary alkalinization before IV contrast protected morphological change protection in rats but did not improve renal function	[64]

AKI, acute kidney injury; Bax, Bcl2-associated X protein; Bcl-2, B-cell lymphoma 2; BUN, blood urea nitrogen; CAT, catalase; CIN, contrast-induced nephropathy; CM, contrast media; Cr, creatinine; CrCl, creatinine clearance; Cyt c, cytochrome c; eNOS, endothelial nitric oxide synthase; ET-1, endothelin-1; FENa, fractional excretion of sodium; GFR, glomerular filtration rate; GSH, glutathione; GSH-Px, glutathione peroxidase; HO-1, heme oxygenase-1; HSA-Trx, recombinant human serum albumin-Thioredoxin-1 fusion protein; ICAM-1; intercellular cell adhesion molecule 1; IL, interleukin; iNOS, inducible nitric oxide synthase; IP, intraperitoneally; IV, intravenously; LC3, light-chain 3; L-NAME, N^ω^-nitro-L-arginine methyl ester; MAPK, mitogen-activated protein kinase; MCP-1, monocyte chemotactic protein-1; MDA, malondialdehyde; MPO, myeloperoxidase; mRNA, messenger ribonucleic acid; MYPT-1, myosin light-chain phosphatase; NAC, N-acetylcysteine; NADPH, nicotinamide adenine dinucleotide phosphate; NAG, N-acetyl-β-glucosaminidase; NF-*k*B, nuclear factor-*k*B; NGAL, neutrophil gelatinase-associated lipocalin; NO, nitric oxide; Nrf-2, Nuclear factor erythroid-derived 2-like 2; OSI, oxidative stress index; PAI-1, plasminogen activator inhibitor 1; PCC, protein carbonyl content; PCR, polymerase chain reaction; PGC-1α, peroxisome proliferator-activated receptor-γ co-activator 1α; Pink1, PTEN-induced putative kinase; RBF, renal blood flow; ROCK-2, Rho kinase 2; RPF, renal plasma flow; RNA, ribonucleic acid; RVR, renal vascular resistance; SC, subcutaneously; SIRT1, sirtuin 1; SOD, superoxide dismutase; TAC, total antioxidant capacity; TBARS, thiobarbituric acid-reacting substances; TGF-1β, transforming growth factor-1β; TNF-α, tumor necrosis factor-α; TOS, total oxidant system; TUNEL, terminal deoxynucleotidyl transferase dUTP nick-end labeling; uKIM-1, urinary kidney injury molecule-1; VEGF, vascular endothelial growth factor; 8-OHdG, 8-hydroxy-2′-deoxyguanosine; ∆ψm, Mitochondrial membrane potential

### Lipid-lowering agents as interventions to reduce CIN

Lipid-lowering agents including rosuvastatin, [30, 31] simvastatin, [31, 32] atorvastatin, [31, 33] xuezhikang (containing lovastatin), [33] and probucol [34] were investigated as potential pharmacological interventions in CIN animal models. These interventions appeared to effectively attenuate CIN as indicated by decreased level of kidney thiobarbiturates (TBARS), serum or renal MDA, serum protein carbonyl content (PCC), and increased serum thiol and glutathione (GSH) [29–34]. Inflammatory markers were also ameliorated as indicated by reduced IL-6, TNF-α, monocyte chemotactic protein-1 (MCP-1), myeloperoxidase (MPO), and increased NO [29–34]. The apoptotic markers were also reduced [29–34]. Furthermore, an appearance of unfavorable histological findings was decreased in an ischemic-reperfusion injury model [29–34]. These findings suggested that statins and probucol could attenuate CIN by modulation of NO, inflammatory responses, oxidative stress and apoptotic processes, leading to improved renal function [29–34]. A summary of these reports on the effects of lipid lowering agents on the protection of CIN is shown in Table 4. There are few clinical studies in this area so these statins are not recommended in the guidelines for CIN prevention.

### Antioxidants as interventions to reduce CIN

Many antioxidants; such as vitamin E, [35] L-carnitine, [36] human serum albumin-thioredoxin-1 fusion protein (HSA-Trx), [37] paricalcitol, [38] N-acetylcysteine (NAC), [39, 40] recombinant manganese SOD (rMnSOD), [41] and agomelatine and melatonin; [42–44] were investigated for their potential effects to prevent CIN in rat models. All of the studies demonstrated the renoprotective effect by attenuating serum Cr and renal histological damage through their antioxidant activities (Table 4). Both inflammatory process and apoptosis were decreased following antioxidant treatments [35–38, 42–44].

Active component of herbs; such as astragaloside, [45] curcumin, [46] grape seed proanthocyanidin, [47] lycopene, [48] magnolin (major active ingredient of herb *Magnolia fargesii*), [49] *Phyllanthus emblica* extract, [50] salidroside, [40] sesame oil, [51] and silymarin, [52] were investigated in CIN in rats (Table 4). All studies demonstrated their benefits in attenuating CIN and AKI biomarkers such as cystatin C, neutrophil gelatinase-associated lipocalin (NGAL), and urine kidney injury molecule-1 (KIM-1), due to reduced oxidative stress and apoptosis.

Other agents such as cardiotrophin-1 and antithrombin III, [53, 54] exendin-4, [55] β-receptor antagonist, [56, 57] phosphodiesterase-5 inhibitor, [58–61] an mTOR inhibitor, [62] exogenous sphingosylphosphorylcholine, [63] and sodium bicarbonate; [64] have been investigated in CIN models (Table 4 and Fig. 3). They all effectively reduced oxidative stress, inflammation and apoptosis, with improved renal histopathology. These findings suggested that these pharmacological interventions prevented CIN through a reduction in oxidative stress, inflammation and apoptosis, leading to improved renal function in rats.

## Pharmacological interventions to reduce CIN: evidence from clinical reports

### Effects of statins on the prevention of CIN

Statins have been shown to exert renoprotective effects in CIN via inhibition of uptake of contrast into renal tubular cells, attenuation of endothelial dysfunction and oxidative stress, anti-inflammation, anti-proliferation of mesangial cells, and protection of podocytes [9]. Clinical studies of statins on the prevention of CIN are summarized in Table 5 and Fig. 3.

**Table 5:** The effects of statins on the prevention of contrast-induced nephropathy: reports from clinical studies

Study type	Models	Intervention (drug/dose/route/duration)	Major findings		Interpretations	References
			Renal function	Oxidative stress/inflammatory markers		
Single-center, double-blind randomized placebo-controlled clinical trial	Age 55–75 years with DM or CKD (Cr > 1.5 mg/dL or GFR 15–60 mL/min/1.73 m^2^) undergoing elective angiography NAC 1200 mg/po/bid/1 day prior to and until 4 h after angiography treated with nonionic iso-osmolar CM	Atorvastatin/80 mg/day/po/48 h prior to angiography (n = 110) vs. Placebo (n = 110)	↓ CIN 24 h after angiography ↔ CIN at 48 h after angiography ↔ Cr	–	Short-term pretreatment with atorvastatin 80 mg along with high-dose NAC decreased incidence of CIN in high-risk patients undergoing angiography	[66]
Prospective, double-blind, randomized, two-arm, parallel group, controlled, clinical trial	Age 18–65 years with Cr 1–1.5 mg/dL or eGFR > 60 mL/min/1.73 m^2^ and controlled DM or hypertension undergoing CAG	Atorvastatin/80 mg/po + NAC/1200 mg/po/OD/3 day prior to and 2 day after angiography (n = 80) vs NAC 1200 mg/po/OD 3 day prior to and 2 day after angiography (n = 80)	Atorvastatin ↓ CIN ↓ mean change in Cr Lesser ↓ eGFR No required dialysis	–	Short-term high-dose atorvastatin along with NAC was effective in prevention of CIN in high risk patients	[67]
Randomized, multicenter, prospective, double-blind clinical trial	Statin-naïve NSTE-ACS undergoing invasive strategy PCI treated with iobitridol	Atorvastatin/80 mg/po/12 h prior to PCI + 40 mg/po/2 h prior to PCI (n = 120) vs. Placebo (n = 121)	↓ CIN ↓ Cr ↓ CrCl change ↓ hospital stay	↓ CRP	Short-term pretreatment with high-dose atorvastatin prevented CIN via anti-inflammatory effects, and shortened hospital stay in patients with ACS undergoing PCI	[68]
Randomized controlled study	Statin-naïve acute STEMI undergoing emergency PCI treated with non-ionic contrast	Atorvastatin/80 mg/po/prior to PCI (n = 78) vs. Placebo (n = 83)	↓ CIN ↓ Cr ↓ cystatin C	–	Short-term pretreatment with high-dose atorvastatin prevented CIN and protected renal function in patients with acute STEMI undergoing emergency PCI	[69]
Prospective, randomized trial	Patients undergoing CAG NAC 600 mg/po/bid/prior to procedure treated with iopamidol	Atorvastatin/80 mg/po/bid/prior to procedure + 80 mg/po/OD/2 day after procedure (n = 60) vs No atorvastatin (n = 70)	↔ CIN ↓ Cr ↑ eGFR ↑ Cr change	–	Short-term atorvastatin protected CIN in patients undergoing CAG	[70]
Randomized trial	CKD (eGFR < 60 mL/min/1.73 m^2^) scheduled for elective CAG or PCI NAC/1200 mg/po/bid/1 day prior to and day of administration of CM treated with iodixanol	Atorvastatin/80 mg/po/24 h prior to iodixanol (n = 202) vs No atorvastatin (n = 208)	↓ CIN ↓ Cr	–	Single high loading dose of atorvastatin administered 24 h before CM exposure was effective in reducing rate of CIN	[29]
Randomized, double-blind, controlled trial	Patients with normal renal function (Cr ≤ 1.5 mg/dL) undergoing elective CTA treated with iopromide	Atorvastatin/80 mg/po/24 h prior to and 48 h after CM (n = 115) vs. Placebo (n = 121)	↔ CIN ↓ Cr	–	Short-term treatment with high dose atorvastatin was effective in reduction of Cr level after CM injection in patients undergoing CTA	[71]
Randomized trial	Patients undergoing CAG	Atorvastatin/10 mg/po/24 h prior to procedure (n = 100) vs Atorvastatin/80 mg/po/24 h prior to procedure (n = 50)	↓ β_2_M ↓ urine NAG/Cr ↑ CrCl All effects by 80 mg > 10 mg	–	Short-term pretreatment with high-dose atorvastatin was superior than low dose on attenuating CIN	[72]
Randomized trial	STEMI undergoing primary PCI treated with iopromide	Atorvastatin/80 mg/po/prior to procedure (n = 98) vs Rosuvastatin/40 mg/po/prior to procedure (n = 94)	↔ CIN ↔ Cr ↔ eGFR ↔ Cr change	–	Short-term pretreatment with atorvastatin or rosuvastatin had similar efficacy in preventing CIN in patients with STEMI undergoing primary PCI	[114]
Prospective, randomized and non-randomized controlled trial	Patients undergoing elective CAG treated with iohexol	Short-term atorvastatin 40 mg/po/3 day prior to and 2 day after CAG (n = 80) No statin (n = 80) Chronic statin therapy/po/at least 1 mo (n = 80) Atorvastatin/10–40 mg/day/po (n = 57) Simvastatin/10–40 mg/day/po (n = 12) Pravastatin/10–20 mg/day/po (n = 6) Rosuvastatin/10 mg/day/po (n = 3) Fluvastatin/80 mg/day/po (n = 2)	↓ Cr (atorvastatin and chronic statin therapy) ↑ GFR (atorvastatin and chronic statin therapy) ↓ cystatin C (chronic statin therapy) ↔ Cr, cystatin C and GFR between short term atorvastatin and chronic statin therapy	–	Short-term and long-term use of atorvastatin had renoprotective effects in low-risk patients undergoing elective CAG	[73]
Observational study	ACS undergoing PCI treated with iopamiron	Simvastatin/40 mg/po/OD/6 months after PCI (n = 128) vs Atorvastatin/20 mg/po/OD/6 months after PCI (n = 143)	↔ Cr ↔ eGFR	–	Simvastatin and atorvastatin were similar renoprotective effects for 6 months after PCI	[115]
Prospective, audited, multicenter regional registry	Patients undergoing PCI	Pre-statin/po (n = 10,831) vs No pre-statin (n = 18,040)	↓ CIN ↓ % of peak Cr ≥ 1.5 mg/dL ↓ nephropathy requiring dialysis	–	Initiating statin therapy before PCI reduced risk of CIN	[65]
Prospective randomized placebo-controlled trial	Patients undergoing CAG treated with iodixanol	Simvastatin/80 mg/day/po/48 h prior to CAG (n = 98) vs. Placebo (n = 96)	↔ GFR in first 24 h ↓ eGFR reduction after 48 h	–	Prophylactic administration of simvastatin reduced CIN	[76]
Prospective, randomized, controlled, multicenter clinical trial	Age 18–75 years with type 2 DM and CKD stage 2–3 undergoing CAG ± PCI treated with iodixanol	Rosuvastatin/10 mg/po/2 day prior to and up to 3 day after procedure (n = 1498) vs No rosuvastatin (n = 1500)	↓ CIN	↓ hsCRP	Short-term rosuvastatin reduced CIN in patients with type 2 DM and CKD undergoing arterial CM injection	[74]
Prospective, randomized trial	Statin-naïve NSTE-ACS patients scheduled for early invasive PCI NAC 1200 mg/po/bid/1 day prior to and 1 day after angiography treated with iodixanol	Rosuvastatin/40 mg/po/prior PCI + 20 mg/po/after PCI (n = 252) vs No rosuvastatin (n = 252)	↓ CIN	–	Short-term high-dose rosuvastatin reduced CIN in statin-naïve NSTE-ACS patients undergoing early invasive PCI	[75]
Randomized trial	ACS undergoing elective PCI treated with iodixanol	Simvastatin/20 mg/po/1 day prior to PCI (n = 115) vs Simvastatin/80 mg/po/1 day prior to PCI (n = 113)	↓ CIN ↓ Cr (80 mg) ↑ CrCl (80 mg)	↓ hsCRP ↓ P-selectin ↓ intercellular adhesion molecule-1	Short-term pretreatment with simvastatin 80 mg before PCI decreased CIN compared with simvastatin 20 mg	[77]
Prospective, single-center, randomized, placebo-controlled trial	CKD (CrCl < 60 mL/min) undergoing elective CAG ± PCI NAC 1200 mg/po/bid/1 day prior to and 1 day after procedure treated with iodixanol	Atorvastatin/80 mg/po/48 h prior to and 48 h after CM (n = 152) vs. Placebo (n = 152)	↔ CIN ↔ Cr ↔ persistent kidney injury	–	Short-term administration of high-dose atorvastatin before and after contrast exposure, in addition to oral NAC, did not decrease CIN occurrence in patients with pre-existing CKD	[79]
Prospective, randomized, double-blind, placebo-controlled, 2-center trial	CKD (CrCl ≤ 60 ml/min ± SCr ≥ 1.1 mg/dl) undergoing CAG	Simvastatin/40 mg/po/every 12 h evening prior to up to morning after procedure (n = 124) vs. Placebo (n = 123)	↔ CIN ↔ Cr ↔ length of hospital stays or 1- and 6-mo	–	Short-term pretreatment with high-dose simvastatin did not prevent CIN in patients with CKD undergoing CAG	[78]
Prospective cohort	CAD ± CKD undergoing CAG	Atorvastatin/10–40 mg/po (n = 1219) vs Rosuvastatin/5–40 mg/po (n = 635)	↔ CIN between 2 groups High plasma atorvastatin or rosuvastatin in CIN subgroups	–	High plasma atorvastatin or rosuvastatin increased risk of CIN	[81]
Retrospective study	Age > 18 years undergoing non-emergent PCI	Statins before PCI (n = 239) Atorvastatin/10–80 mg/po (n = 89) Simvastatin/10–80 mg/po (n = 74) Pravastatin/10–40 mg/po (n = 53) Lovastatin/20–40 mg/po (n = 13) Rosuvastatin/5–20 mg/po (n = 9) Fluvastatin/po (n = 1) No statin before PCI (n = 114)	↑ CIN	–	Statin use before non-emergent PCI increased incidence of CIN	[80]

ACS, acute coronary syndrome; β_2_M, β_2_-microglobulin; CAD, coronary artery disease; CAG, coronary angiography; CIN, contrast-induced nephropathy; CKD, chronic kidney disease; CM, contrast media; Cr, creatinine; CrCl, creatinine clearance; CRP, C-reactive protein; CTA, computed tomography angiography; DM, diabetes mellitus; eGFR, estimated glomerular filtration rate; GFR, glomerular filtration rate; hsCRP, high-sensitivity C-reactive protein; NAC, N-acetylcysteine; NAG, NAG, N-acetyl-β-glucosaminidase; NSTE-ACS, non-ST-elevated acute coronary syndrome; PCI, percutaneous coronary intervention; STEMI, ST-segment elevation myocardial infarction

In a retrospective study of 29,409 patients undergoing percutaneous coronary intervention (PCI), initiating statin therapy before PCI reduced risk of CIN [65]. Many randomized-controlled trials of atorvastatin for CIN prevention were done in patients undergoing coronary angiography (CAG). In high risk patients, short-term pretreatment with high-dose atorvastatin decreased incidence of CIN [29, 66–73], reducing C-reactive protein (CRP) [68].

The largest randomized-controlled trial in rosuvastatin was done in 2,998 patients with type 2 DM and CKD who underwent coronary or peripheral angiography, receiving either pre and post-intervention rosuvastatin or standard care. The rosuvastatin-treated group had lower incidence of CIN and high-sensitivity CRP (hsCRP) [74]. In the PRATO-ACS trial, the incidence of CIN in non-ST elevated ACS patients undergoing CAG who receive rosuvastatin in statin-naïve patients was lower than in control group [75]. With simvastatin, the prospective randomized-controlled trials in patients undergoing CAG demonstrated that short-term pretreatment of high-dose simvastatin reduced the incidence of CIN [76, 77]. Simvastatin also reduced inflammation by decreasing hsCRP, P-selectin, and intracellular cell adhesion molecule 1 (ICAM-1) [77].

Despite these promising findings, inconsistent reports exist. The PROMISS trial failed to show a difference between simvastatin and placebo with respect to a primary end point based on the mean peak increase in plasma Cr within 48 h after CAG in patients with CKD [78]. Also, another randomized-controlled trial demonstrated that short-term administration of high-dose atorvastatin with oral NAC did not decrease incidence of CIN in pre-existing CKD patients, [79] and a retrospective study, statin given before non-emergent PCI increased the incidence of CIN [80]. Similarly, a prospective cohort study in patients with or without CKD undergoing CAG demonstrated that high plasma atorvastatin or rosuvastatin was associated with increased CIN risk [81]. Therefore, currently statins are not recommended in the guidelines for CIN prevention.

### Effects of antioxidants on the prevention of CIN

Many clinical trials have investigated the effects of various antioxidants on the prevention of CIN. These include NAC, ascorbic acid, sodium bicarbonate, sodium-2-mercaptoethane sulphonate (MESNA), and nebivolol. A summary of these reports is shown in Table 6 and Fig. 3.

**Table 6:** The effects of non-statin on the prevention of contrast-induced nephropathy: reports from clinical studies

Study type	Models	Intervention (drug/dose/route/duration)	Major findings		Interpretations	References
			Renal function	Oxidative stress/inflammatory markers		
Randomized, double-blind, placebo-controlled trial	Patients with Cr ≥ 1.2 mg/dL undergoing clinically driven, nonemergent CAG or PCI treated with nonionic, low- or iso-osmolar contrast	Ascorbic acid/3 g/po/2 h prior to procedure + 2 g/po/night and morning after procedure (n = 118) vs. Placebo (n = 113)	↓ CIN ↓ Cr ↓ CrCl changes ↔ BUN	–	Ascorbic acid prevented CIN after coronary imaging procedures in patients with pre-existing renal dysfunction	[110]
Prospective randomized-controlled trial	Patients with Cr > 1.2 mg/dL or CrCl < 50 mL/min underwent elective CT treated with iopromide	NAC/600 mg/po/bid/1 day prior to and after CT (n = 41) vs. Placebo (n = 42)	↓ CIN ↓ Cr changes at 48 h after CT	–	Short-term pretreatment with NAC prevented CIN	[82]
Prospective randomized-controlled trial	Patients with Cr > 1.2 mg/dL or CrCl < 70 mL/min underwent elective CAG ± PCI treated with iopromide	NAC/600 mg/po/bid/1 day prior to and after CAG (n = 92) No NAC (n = 91)	↔ CIN ↔ Cr changes at 48 h after CAG ↓ Cr changes at 48 h after CAG by using small volume of CM	–	Short-term NAC prevented CIN in patients with CKD and using small volume of CM	[83]
Randomized, double-blind, placebo-controlled trial	Patients with Cr ≥ 1.4 mg/dL or CrCl < 50 mL/min underwent elective CAG treated with ioxilan	NAC/600 mg/po/bid/1 dose prior to and 3 doses after CAG (n = 25) vs. Placebo (n = 29)	↓ CIN ↓ Cr at 48 h after CAG ↓ Cr changes	–	Short-term NAC reduced risk of CIN in patients with CKD	[84]
Prospective randomized, double-blind study	Patients with Cr > 106 µmol/L underwent elective CAG treated with non-ionic, low osmolar iodine	NAC/1,000 mg/po/bid/24 h prior to and 24 h after CAG (n = 24) vs. Placebo (n = 25)	↓ CrCl changes at 24 and 96 h after CAG	↑ urinary NO ↔ urinary F2-isoprostanes	Short-term NAC prevented CIN in patients with CKD undergoing CAG via increasing NO production	[85]
Prospective, randomized, double-blind, placebo-controlled trial	Patients with Cr > 1.2 mg/dL or CrCl < 60 mL/min underwent elective CAG ± PCI treated with iopamidol	NAC/600 mg/po/bid/1 day prior to and after procedure (n = 102) vs. Placebo (n = 98)	↓ CIN ↓ Cr at 48 h after procedure ↑ CrCl	–	Short-term NAC prevented CIN in patients with moderate CKD after CAG	[86]
Prospective randomized trial	Patients with Cr ≥ 1.5 mg/dL underwent CAG treated with iopromide or ioxilan	NAC/600 mg/po/bid/after randomization, 4 h later and every 12 h after CAG total 5 doses (n = 21) vs. Placebo (n = 22)	↓ CIN ↓ Cr changes at 48 and 72 h after CAG	–	Short-term NAC reduced CIN in patients with mild to moderate renal impairment undergoing CAG	[87]
Prospective randomized trial	Patients with Cr > 1.8 mg/dL (males), > 1.6 mg/dL (females), or CrCl < 50 mL/min underwent CAG ± PCI	NAC/1000 mg/po/bid/1 h prior to and 4 h after procedure (n = 36) vs. Placebo (n = 44)	↔ CIN ↓ Cr changes at 48 h	–	Short-term high-dose NAC prevented the rise of Cr 48 h after CAG/PCI and might prevent CIN	[88]
Prospective randomized-controlled trial	Patients with Cr > 2.0 mg/dL and < 6.0 mg/dL or CrCl < 40 mL/min and > 8 mL/min underwent CAG treated with iopamiro	NAC/400 mg/po/bid/1 day prior to and after CAG (n = 60) vs. Placebo (n = 61)	↓ Cr ↓ Cr changes at 48 h	–	Short-term NAC protected CIN in patients with CKD undergoing CAG	[89]
Prospective randomized-controlled trial	Patients with eGFR 30–60 mL/min/1.73 m^2^ underwent CAG treated with ioversol	NAC/600 mg/po/bid/1 day prior to and after CAG (n = 73) vs NAC/600 mg/po/bid/1 day prior to and after CAG + theophylline/200 mg/po/bid/1 day prior to and after CAG (n = 72) vs No NAC (n = 72)	↓ CIN (NAC + theophylline) ↓ Cr at 48 h after CM (NAC + theophylline)	–	Short-term NAC along with theophylline prevented CIN in patients with eGFR 30–60 mL/min/1.73 m^2^	[90]
Double-blind, placebo-controlled, randomized study	Age 18–80 years with Cr 1.4–5.0 mg/dL and CrCl < 70 mL/min/1.73 m^2^ scheduled for elective CAG treated with iopamidol	NAC/600 mg/po/bid/2 day prior to and 2 day after angiography (n = 13) vs. Placebo (n = 11)	↑ CrCl ↓ α-GST	↔ urinary 15-isoprostane F2_t_	Short-term NAC treatment was associated with suppression of oxidative stress-mediated proximal tubular injury	[91]
Prospective randomized-controlled trial	Patients with Cr > 1.36 mg/dL or CrCl < 50 mL/min underwent CAG or PCI treated with iodixanol	NAC/150 mg/kg/IV/30 min prior to CM + NAC/50 mg/kg/IV/4 h after CM (n = 41) vs No NAC (n = 39)	↓ CIN ↓ Cr at 48 and 96 h after CM	–	Short-term IV NAC prevented CIN	[107]
Single center, Prospective, single-blind, placebo-controlled, randomized controlled trial	STEMI undergoing primary PCI treated with iopromide	NAC/1200 mg/day/IV/bid/bolus prior to and up to 48 h after PCI (n = 126) vs. Placebo (n = 125)	↔ CIN ↔ Cr ↔ CrCl	↓ activated oxygen protein products at day 1–2 ↓ oxidized LDL at day 1–3	High-dose IV NAC reduced oxidative stress after reperfusion of MI but not provided additional clinical benefit to nephropathy	[108]
Randomized, placebo-controlled, double blind trial	Age > 18 years with Cr ≥ 1.2 mg/dL or CrCl < 50 mL/min underwent CAG treated with iomeperole	NAC/600 mg/po/bid/1 day prior to and after CAG (n = 19) vs Zinc/60 mg/po/1 day prior to CAG (n = 18) vs. Placebo (n = 17)	↔ CIN ↔ Cr ↓ cystatin C	–	Short-term NAC and zinc did not prevent CIN but NAC had renoprotective effect by reducing cystatin C	[92]
Double-blind, placebo and comparator-drug-controlled, randomized trial	eGFR 15–44.9 mL/min/1.73 m^2^ or 45–59.9 mL/min/1.73 m^2^ in DM underwent CAG or noncoronary angiography	NAC/1200 mg/po/bid/1 h prior to, 1 h, and 4 day after angiography (n = 2495) vs. Placebo (n = 2498)	↔ CIN ↔ Cr at 90–104 day after angiography	–	Oral NAC did not prevent CIN	[93]
Pragmatic randomized-controlled trial	Patients with at least 1 risk factor for CIN (age > 70 years, Cr > 1.5 mg/dL, DM, CHF, LVEF < 0.45, hypotension) underwent coronary or peripheral arterial diagnostic intravascular angiography or percutaneous intervention	NAC/600 mg/po/bid/1 day prior to and after procedure (n = 1172) vs. Placebo (n = 1136)	↔ CIN ↔ Cr	–	Short-term NAC did not reduce the risk of CIN	[94]
Randomized prospective study	Patients with Cr ≥ 1.6 mg/dL or CrCl ≤ 60 mL/min underwent PCI treated with low-osmolality nonionic CM	NAC/600 mg/po/bid/1 day prior to and after procedure (n = 45) vs Fenoldopam/0.1 µg/kg/min/IV/4 h prior to and 4 h after procedure (n = 38) vs No NAC or fenoldopam (n = 40)	↔ CIN ↔ Cr changes at 24 and 48 h after procedure	–	Short-term NAC or fenoldopam did not prevent CIN in patients with CKD	[95]
Prospective, double-blind, placebo-controlled, randomized clinical trial	Age > 18 years with DM and Cr ≥ 1.5 mg/dL for men and ≥ 1.4 mg/dL for women underwent elective CAG treated with iohexol or iodixanol or diatrizoate meglumine	NAC/600 mg/po/bid/24 h prior to and after procedure (n = 45) vs. Placebo (n = 45)	↔ CIN ↔ Cr changes at 48 after CAG ↔ BUN changes at 48 after CAG ↔ CrCl changes at 48 after CAG	–	Short-term NAC did not prevent CIN in patients with DM and CKD	[96]
Prospective randomized-controlled trial	Patients with Cr > 1.2 mg/dL or CrCl < 50 mL underwent elective CAG treated with iodixanol	NAC/600 mg/po/bid/1 day prior to and after CAG (n = 73) No NAC (n = 106)	↔ CIN ↔ Cr changes at 48 h after CAG	–	Short-term NAC did not prevent CIN in patients with CKD	[97]
Randomized-controlled trial	Patients with Cr > 1.7 mg/dL underwent CAG treated with iohexol	NAC/1200 mg/po/1 h prior to and 3 h after CAG (n = 38) vs. Placebo (n = 41)	↔ CIN ↔ Cr changes at 48 h after CAG	–	Short-term NAC did not prevent CIN after CAG	[98]
Prospective, randomized clinical study	Age ≥ 18 years with CrCl < 55 ml/min underwent elective coronary ± peripheral angiography treated with iodixanol	NAC/600 mg/po/bid/1 day prior to and after procedure (n = 99) vs. Placebo (n = 101)	↔ CIN	–	Short-term NAC did not prevent CIN	[99]
Prospective, open-label, randomized, controlled trial	Patients with Cr 1.69–4.52 mg/dL underwent elective CAG or PCI treated with iopromide	NAC/400 mg/po/tid/1 day prior to and after procedure (n = 46) vs No NAC (n = 45)	↔ CIN ↔ Cr changes at 48 h after procedure ↔ eGFR changes at 48 h after procedure	–	Short-term NAC did not prevent CIN in patients with moderate to severe renal insufficiency undergoing CAG or PCI	[100]
Multicenter, randomized, double-blind, placebo-controlled clinical trial	Diabetic patients with Cr ≥ 106.08 µmol/L or CrCl < 50 mL/min underwent elective CAG or PCI treated with ioxaglate	NAC/600 mg/po/bid/1 day prior to and after procedure (n = 77) vs. Placebo (n = 79)	↔ CIN ↔ Cr changes at 48 h after procedure ↔ CrCl changes at 48 h after procedure	–	Short-term NAC did not prevent CIN in patients undergoing cardiac catheterization	[101]
Prospective, randomized, double-blind placebo-controlled trial	Patients with Cr ≥ 1.5 mg/dL or CrCl < 50 mL/min underwent CAG treated with iopamidol	NAC/600 mg/po/tid/24 h prior to and after procedure (n = 41) vs. Placebo (n = 39)	↔ CIN	–	Short-term NAC did not prevent CIN in CKD patients undergoing CAG	[102]
Prospective, randomized, double-blind, placebo-controlled trial	Age ≥ 19 years with Cr > 1.2 mg/dL and CrCl < 50 mL/min underwent elective CAG ± PCI treated with iopamidol	NAC/1,500 mg/po/1 day prior to and every 12 h after procedure for 4 doses (n = 49) vs. Placebo (n = 47)	↔ CIN ↔ Cr ↔ BUN	–	Short-term NAC did not prevent CIN in patients with CKD undergoing elective CAG	[103]
Prospective, randomized, single-blinded, single-center clinical trial	Age > 18 years with eGFR > 30 mL/min/1.73 m^2^ underwent elective CAG or PCI treated with iopromide	NAC/600 mg/po/bid/24 h prior to and after procedure (n = 157) vs NaHCO_3_/1.5 mL/kg/h/IV/6 h prior to and 6 h after procedure (n = 159) vs. NAC/600 mg/po/bid/24 h prior to and after procedure + NaHCO_3_/1.5 mL/kg/h/IV/6 h prior to and 6 h after procedure (n = 150) vs No NAC or NaHCO_3_ (n = 161)	↔ CIN	–	NAC and NaHCO3 did not reduce incidence of CIN	[104]
Single-center prospective controlled trial	Patients with Cr > 1.2 mg/dL underwent CAG or PCI treated with ioxaglate	NAC/600 mg/po/bid/1 day prior to and after procedure (n = 88) vs NaHCO_3_/1 mL/kg/h/IV/6 h prior to and 6 h after procedure (n = 88) vs No NAC or NaHCO_3_ (n = 88)	↓ CIN (NaHCO_3_ > NAC > No NAC or NaHCO_3_) ↓ CrCl (NaHCO_3_ > NAC = No NAC or NaHCO_3_)	–	NaHCO_3_ protected CIN better than NAC and standard treatment	[105]
Prospective randomized trial	Patients with CrCl > 30 mL/min/1.73 m^2^ underwent CAG ± PCI treated with iopromide	NAC/1200 mg/IV/12 h prior to and after procedure (n = 53) vs. Placebo (n = 51)	↔ CIN ↔ CrCl	–	Short-term IV NAC did not prevent CIN in patients with normal, mild and moderate CKD undergoing coronary procedure	[109]
Single center, prospective, randomized study	CAD with Cr ≥ 1.5 mg/dL ± CrCl < 60 mL/min) who underwent elective CAG treated with iomeprol	NAC/704 mg/po/bid/1 day prior to and up to 2 day after CAG (n = 7) vs GSH/100 mg/min/IV/30 min prior to CAG (n = 7) vs Control group (n = 7)	↔ CIN	↑ LOOHs at 2 h after CAG (control > NAC > GSH) ↓ serum GSH at 2 h after CAG (NAC > control > GSH)	GSH protected kidney against CM-induced oxidative stress more effectively than oral administration of NAC before CAG	[106]
Randomized trial	Age > 18 years underwent elective or emergent CAG	NaHCO_3_ (166 mEq/L)/3 mL/kg/h/IV/1 h prior to CAG + 1 mL/kg/h/IV/6 h after CAG (n = 50) vs NaHCO_3_ (166 mEq/L)/3 ml/kg/h/IV/6 h prior to CAG + 1 mL/kg/h/IV/6 h after CAG (n = 50)	↑ Cr and ↓ eGFR 48 h post-intervention (short regimen) ↔ Cr and↔ eGFR 48 h post-intervention (long regimen) ↓ serum K	–	Long-term regimen of bicarbonate supplementation was more effective strategy to prevent CIN than short regimen	[111]
Cross-sectional case–control study	CAD with at least 1 risk factor for CIN (DM, advanced age, reduced GFR, anemia) undergoing CAG	Nebivolol/po/at least 1 mo (n = 45) vs No nebivolol (n = 114)	↔ CIN ↔ Cr, eGFR, NGAL in both groups before and after CAG ↑ Cr and NGAL and ↓ eGFR in both groups compared to levels before CAG	–	Nebivolol did not prevent CIN in patients undergoing CAG	[113]
Pilot study	Patients with Cr > 2 mg/dL undergoing CAG treated with iomeprol	MESNA/800 mg/IV/30 min prior to and up to 4 h after iomeprol (n = 12)	↓ CIN ↓ Cr at 48 h	–	MESNA prevented CIN in patients with renal impairment	[112]

α-GST, α-glutathione S-transferase; BUN, blood urea nitrogen; CAD, coronary artery disease; CAG, coronary angiography; CHF, congestive heart failure; CIN, contrast-induced nephropathy; CKD, chronic kidney disease; CM, contrast media; Cr, creatinine; CrCl, creatinine clearance; CT, computed tomography; DM, diabetes mellitus; eGFR, estimated glomerular filtration rate; GFR, glomerular filtration rate; GSH, glutathione; IV, intravenously; LDL, low-density lipoprotein; LOOHs, lipid hydroperoxides; LVEF, left ventricular ejection fraction; MI, myocardial infarction; NAC, *N*-acetylcysteine; NGAL, neutrophil gelatinase-associated lipocalin; NO, nitric oxide; PCI, percutaneous coronary intervention; STEMI, ST-segment elevation myocardial infarction

#### *N*-Acetylcysteine (NAC)

For nearly two decades, many randomized-controlled trials have investigated the roles of oral NAC for CIN prevention in patients receiving PCI or diagnostic CAG undergoing computed tomography. They demonstrated the protective effect of NAC to CIN in both low and high risk patients compared with placebo [82–91]. The prospective studies demonstrated that NAC prevented the decline in urinary NO end-products [85, 91]. However, many conflicting reports exist, with no significant evident benefits of oral NAC in CIN prevention [92–106]. Therefore, routine administration of NAC for the prevention of CIN or longer-term adverse events after angiographic procedures is not recommended [93].

Decisive factors in NAC for CIN prevention are dosage and treatment duration. NAC was commonly given only for two days prior to CM administration in those trials. It is possible that the duration of NAC treatment was too short to be effective in counteracting CIN-induced ROS production. Furthermore, since NAC has a very short plasma half-life, dosing twice daily could be insufficient to achieve consistent renal protective effects [7]. Future studies are needed to test this hypothesis. A summary of these reports on the effects of oral administration of NAC to prevent CIN is shown in Table 6.

Effects of intravenous administration of NAC on CIN protection has been investigated in patients requiring emergent CAG. In a prospective randomized-controlled study in low risk patients, short-term intravenous NAC treatment could prevent CIN [107]. However, 7% of the patients developed anaphylactoid reactions in that report. In addition, conflicting reports exist [108, 109]. Intravenous NAC reduced oxidative stress after reperfusion of myocardial infarction, however it did not provide additional clinical benefit to nephropathy [108, 109]. Also, intravenous NAC at higher doses could be associated with significant side effects (anaphylactoid reaction, hypotension, bronchospasm) [12]. Despite equipoise on its efficacy, NAC has been widely used in clinical practice in high risk patients due to its low cost, ready availability, easy administration, and limited toxicity in an oral form.

#### Ascorbic acid, sodium bicarbonate and sodium-2-mercaptoethane sulphonate (MESNA)

In a randomized, double-blind, placebo-controlled trial of patients with Cr ≥ 1.2 mg/dL undergoing CAG, the use of ascorbic acid was associated with a significant reduction in the rate of CIN [110]. For sodium bicarbonate, a randomized trial in patients undergoing CAG demonstrated that bicarbonate supplementation prevented CIN when it was given 6 h prior to CAG and administered continuously for another 6 h after CAG [111]. MESNA has the potential to act as a ROS scavenger. In a pilot study in CKD patients undergoing CAG, renal function improved after MESNA pre and posttreatment [112]. These findings indicated that these antioxidants could prevent CIN in patients with renal impairment. However, future studies may add weight to these limited reports.

#### Nebivolol

Although in vivo studies report the benefits of antioxidant effects by β-receptor antagonists leading to prevention of CIN, [56, 57] a report from a clinical study demonstrated otherwise. In a cross-sectional case–control study, the patients with risk factor for CIN that received nebivolol had no change in renal function before and after CAG, and did not prevent CIN in patients undergoing CAG [113]. These inconsistent reports could be due to a small sample size from a single center and different times of follow-up on Cr since a 1 month follow up might allow the development of a tolerance to the vasodilatory effects of the drug.

In summary, this review provides a comprehensive narrative review that summarizes findings of the pathogenesis of CIN from in vitro and in vivo studies and the novel interventions for prevention of CIN. Consistent as well as controversial reports regarding the clinical findings are also summarized for potential interventions to prevent CIN. This review provides fundamental knowledge for future basic and clinical studies to find novel interventions to prevent CIN in a clinical setting. However, since our review does not include the non-English original articles it is possible that other potential interventions to prevent CIN be missing.

## Conclusions

CIN is associated with adverse outcomes. These include renal replacement therapy, prolonged hospitalization, increased mortality, and increased financial burden. For this reason, the appropriate prophylactic interventions before CM administration in high-risk patients are important in reducing CIN. Since hypoxic-toxic injury, including altered renal microcirculation, medullary hypoxia and ROS-mediated cellular injury is a fundamental pathogenesis of CIN, understanding these various complex pathways could lead to prevention. Although a variety of experimental studies and clinical trials have demonstrated potential pharmacological interventions to prevent CIN, inconclusive results exist. Future double-blinded randomized-controlled trials with large populations of oral or intravenous antioxidants as well as other novel compounds are needed to warrant their use to prevent CIN in patients exposed to CM.

## Data Availability

Not applicable.

## Abbreviations

AKI: Acute kidney injury
ATF2: Activating transcriptional factor 2
Bax: B-cell lymphoma 2-associated X protein
Bcl-2: B-cell lymphoma-2
CAG: Coronary angiography
CI-AKI: Contrast-induced acute kidney injury
CIN: Contrast-induced nephropathy
CKD: Chronic kidney disease
CM: Contrast media
Cr: Creatinine
ERK: Extracellular signal-related kinases
ESRD: End-stage renal disease
GSH: Glutathione
HO-1: Heme oxygenase 1
HSA-Trx: Human serum albumin-thioredoxin-1 fusion protein
hsCRP: High-sensitivity C-reactive protein
ICAM-1: Intracellular cell adhesion molecule 1
IL: Interleukin
JNK: C-JUN N-terminal kinase
KDIGO: Kidney Disease Improving Global Outcomes
KIM-1: Kidney injury molecule-1
MAPK: Mitogen-activated protein kinase
MCP-1: Monocyte chemotactic protein-1
MDA: Malondialdehyde
MESNA: Sodium-2-mercaptoethane sulphonate
MPO: Myeloperoxidase
NAC: *N*-Acetylcysteine
NAD: Nicotinamide adenine dinucleotide
NF-*k*B: Nuclear factor-*k*B
NGAL: Neutrophil gelatinase-associated lipocalin
NO: Nitric oxide
Nox: Nicotinamide adenine dinucleotide phosphate oxidase
Nrf-2: Nuclear factor erythroid 2-related factor 2
PCC: Protein carbonyl content
PCI: Percutaneous coronary intervention
PGC-1α-FoxO1: Peroxisome proliferator-activated receptor gamma-assisted activating factor-1α-Forkhead-box transcription factor 1
rMnSOD: Recombinant manganese superoxide dismutase
ROCK: Rho-kinase
ROS: Reactive oxygen species
siRNA: Short interfering ribonucleic acid
SIRT1: Silent information regulator 1
SOD: Superoxide dismutase
TBARS: Thiobarbiturates
TNF-α: Tumor necrosis factor-α
